# Volcanic Vortex Rings: Axial Dynamics, Acoustic Features, and Their Link to Vent Diameter and Supersonic Jet Flow

**DOI:** 10.1029/2021GL092899

**Published:** 2021-07-28

**Authors:** J. Taddeucci, J. J. Peña Fernández, V. Cigala, U. Kueppers, P. Scarlato, E. Del Bello, T. Ricci, J. Sesterhenn, S. Panunzi

**Affiliations:** ^1^ Istituto Nazionale di Geofisica e Vulcanologia Rome Italy; ^2^ Ludwig‐Maximilians‐Universität München Munich Germany; ^3^ Universität Bayreuth Bayreuth Germany

**Keywords:** vortex ring, jet noise, vent diameter, Strombolian, Stromboli

## Abstract

By injecting a mixture of gas and pyroclasts into the atmosphere, explosive volcanic eruptions frequently generate vortex rings, which are toroidal vortices formed by the jet's initial momentum. Here, we report high‐speed imaging and acoustic measurements of vortex rings sourcing from gas‐rich eruptive jets at Stromboli volcano (Italy). Volcanic vortex rings (VVRs) form at the vent together with an initial compression acoustic wave, VVRs maximum rise velocity being directly proportional to the amplitude and inversely proportional to the duration of the compression wave. The axial rise and acoustic signature of VVRs match well those predicted by recent fluid‐dynamic experiments. This good match allows using the high‐frequency (80–1,000 Hz) component of the jet sound and the time‐dependent rise of VVRs to retrieve two key eruption parameters: the Mach number of the eruptive jets (<1.5) and vent diameter (∼0.7 m), respectively, the latter being confirmed independently by direct Uncrewed Aerial Vehicle observations.

## Key Points

- Volcanic vortex rings are formed by gas‐rich, jet‐forming Strombolian‐style explosions
- The explosion acoustic signals bear the signature of the vortex rings and reveal supersonic eruptive jets with Mach number up to 1.5
- Vent diameter can be estimated from the time‐dependent rise of vortex rings at 0.7 m, in agreement with direct observation from Uncrewed Aerial Vehicle

## 1 Introduction

Subaerial explosive volcanic eruptions typically involve the rapid injection of a mixture of gas and particles (pyroclasts) from a volcanic vent into a relatively static atmosphere (Sigurdsson, 2015). A jet entering a static fluid from a nozzle is well known to generate a vortex ring, that is, a toroidal vortex formed by the fluid roll‐up at the nozzle lip and absorbing part of the jet's initial momentum (e.g., Lamb, 1945; Lim & Nickels, 1995; Shariff & Leonard, 1992). Vortex rings are peculiar, relatively stable fluid‐dynamic features, and have specific translation and acoustic signatures that depend on the source jet and nozzle properties (e.g., Brouillette & Herbert, 1997; Gharib et al., 1998; Peña Fernández & Sesterhenn, 2017, 2019; Peña Fernández et al., 2020).

Vortex rings generated by volcanic activity (hereafter volcanic vortex rings, or VVRs) have been documented for a long time. Among the first VVRs reported there are smoke rings formed during eruptions by impulsive degassing events or short explosions (Fuentes, 2014; Perret, 1912). More recently, VVRs associated with eruptive jets have often been reported at ash‐rich, short‐lived, transient eruptions of Vulcanian and Strombolian styles (Chojnicki et al., 2015; Clarke et al., 2015; Patrick, 2007; Tournigand et al., 2017). For such eruptions, the rise velocity of VVRs has been used to draw inferences on a number of eruption and source parameters, including temperature, particle content, magma discharge rate, and exit velocity of the eruption mixture at the vent, as well as on the aerodynamic behavior of individual pyroclasts within the jet (Patrick, 2007; Sparks & Wilson, 1982; Suwa et al., 2014; Taddeucci et al., 2015; Tournigand et al., 2017, 2019).

Parallels between eruptive volcanic jets and those produced by shock‐tube experiments have been identified (Bennett, 1971, 1974; McGetchin & Ulrich, 1973; Shoemaker et al., 1962), also with specific reference to vortex ring dynamics (Kieffer & Sturteivant, 1984). More recently, experimental investigations of volcanic jets and vortex rings focused on air entrainment and unsteady jet evolution (Chojnicki et al., 2014, 2015), particle acceleration and ejection (Cigala et al., 2017; Salvatore et al., 2020), and acoustic emissions from transient eruptions (Peña Fernández et al., 2020). Here, we provide the first joint parameterization of the motion and acoustic emissions of VVRs by synchronized high‐speed imaging and audible‐to infrasonic‐band acoustic recordings. The target of the study was VVRs formed by gas‐rich eruptive jets from Strombolian activity. Leveraging new, well‐constrained theoretical and experimental models of vortex rings propagation and sound formation, VVRs parameters are used to derive two key eruption parameters, the Mach number of the eruptive jets and the diameter of the eruptive vent.

## 2 Materials and Methods

We measured VVRs from gas‐rich, jet‐forming Strombolian explosions at Stromboli volcano (Italy) on July 12, 2018 and, with poorer visibility, on May 11, 2019. All explosions occurred at the same vent, located in the SW vent area. Ten and seven explosions were recorded in 2018 and 2019, respectively. Most of the 2018 explosions produced multiple VVRs, and a total of 30 VVRs were measured, of which 26 were suitable for analysis.

The explosions were documented visually by using an Optronis CR600X2 high‐speed camera (1,280 × 1,024 pixels), filming continuously for 30 s at 500 or 1,000 frames per second. At filming distances of 331 and 551 m, the pixel pitch of the high‐speed camera was 0.012 and 0.019 m in 2018 and 2019, respectively. Acoustic signals were recorded with a GRAS 40AN microphone (frequency range (±2 dB): 0.5–20 kHz, sensitivity @ 250 Hz (±1 dB): 50 mV/Pa, dynamic range up to 149 dB or 564 Pa), acquired at a rate of 10 kHz with a Slomotech Highsync 1250 data logger. None of the recorded signals are clipped.

**Figure 1:**
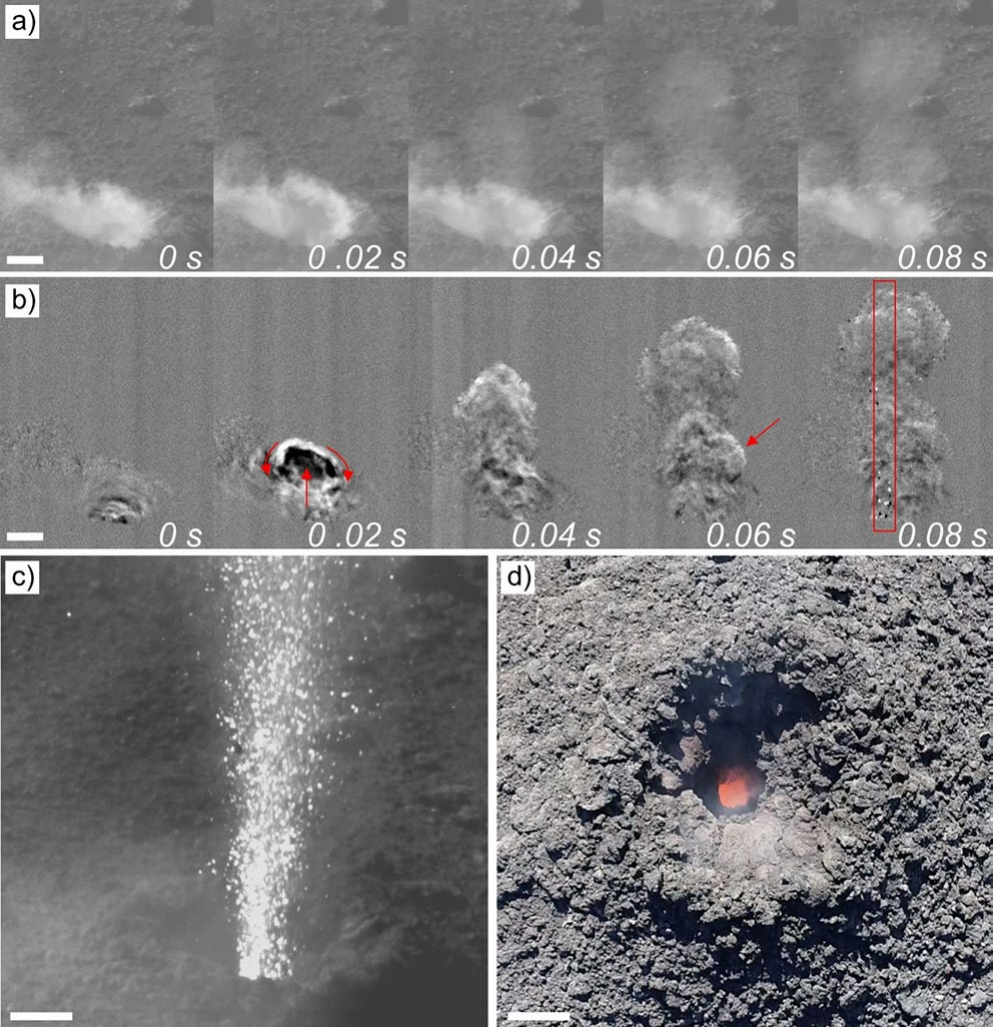
Volcanic vortex ring from a Strombolian explosion. (a) Original still frames (cropped and decimated) from a high‐speed video. (b) Image difference of the same frames in (a). In red, the motion of the vortex ring at 0.02 s, a large trailing vortex at 0.06 s, and the swath area used for the rise diagrams at 0.08 s (c) A jet of incandescent pyroclasts (in white) leaving the vent 1 s after the vortex ring. (d) Aerial view of the source vent of the volcanic vortex rings in 2018. Scale bar is 1 m long in all images.

After performing image difference (i.e., frame‐by‐frame subtraction) of the original high‐speed videos to remove the static background and highlight moving objects (Figure 1), the position of VVRs over time has been measured every one frame, with an error of ±2 pixels and <1 μs in space and time, respectively, both by manual tracking and by their slope in rise diagrams. These diagrams are obtained by averaging row‐wise the gray level values of pixels in a swath centered on the vent and plotting the corresponding vector of pixel values over time for each frame (Gaudin et al., 2017). The obtained plot displays graphically the vertical motion of VVRs and pyroclasts over time, with a stripe whose slope is proportional to their vertical velocity (cf. Delle Donne & Ripepe, 2012; Gaudin et al., 2017). We also recorded, both by manual tracking and by their slope in rise diagrams, the exit velocity of pyroclasts ejected in the first 0.1 s after the VVR appearance, as well as the time interval between the appearance of the VVR and pyroclasts.

The time‐frequency evolution of the acoustic signals has been analyzed by using a complex Morlet wavelet transformation as per Peña Fernández et al. (2020). Differently from spectrograms based on short‐time Fourier transform (STFT), wavelet scalograms have a constant relative error in the frequency space, favoring feature visualization over the whole frequency range investigated (see comparison in Figure S1). The amplitude and duration of the first acoustic peak generated by VVRs have been measured using the “prominence” and “width at half prominence” features of the Matlab^®^R2020b built‐in “findpeaks” function. Peak duration has been considered as the double of peak width at half prominence. To account for the different locations of the 2018 and 2019 measurements, the peak amplitude of the 2019 explosions has been reduced to that of a recording distance of 331 m from the vent (same distance as the 2018 measurements) using Equation 1 of Johnson and Ripepe (2011).

Aerial views of the active vent directly from above were collected with a DJI Phantom 4 Pro + Uncrewed Aerial Vehicle (UAV).

## 3 Visual and Acoustic Features of Volcanic Vortex Rings

The recorded explosions were gas‐dominated Type 0 events (Leduc et al., 2015) that formed jets sustained for up to 1 min, occasionally interrupted by a few, short (subsecond) pauses. Each jet started with the formation of a VVR followed by the ejection of incandescent pyroclasts after a variable time interval (Figure 1). In 2018, pauses were longer and more abundant than in 2019, with multiple VVRs being formed by short gas ejection pulses at the beginning of some explosions and when jetting restarted after longer pauses. In side view, VVRs display a roughly elliptical shape, with clearly counter‐rotating outer edges (Figure 1). Aerial observations of the active vent revealed, both in 2018 and 2019, an approximatively circular orifice, funnel‐shaped, 0.8–0.7 m in diameter in its narrower visible part and ∼3.5 m at the outer rim, in good agreement with the smallest diameter of the eruptive jet of pyroclasts (0.9 m, Figure 1).

**Figure 2:**
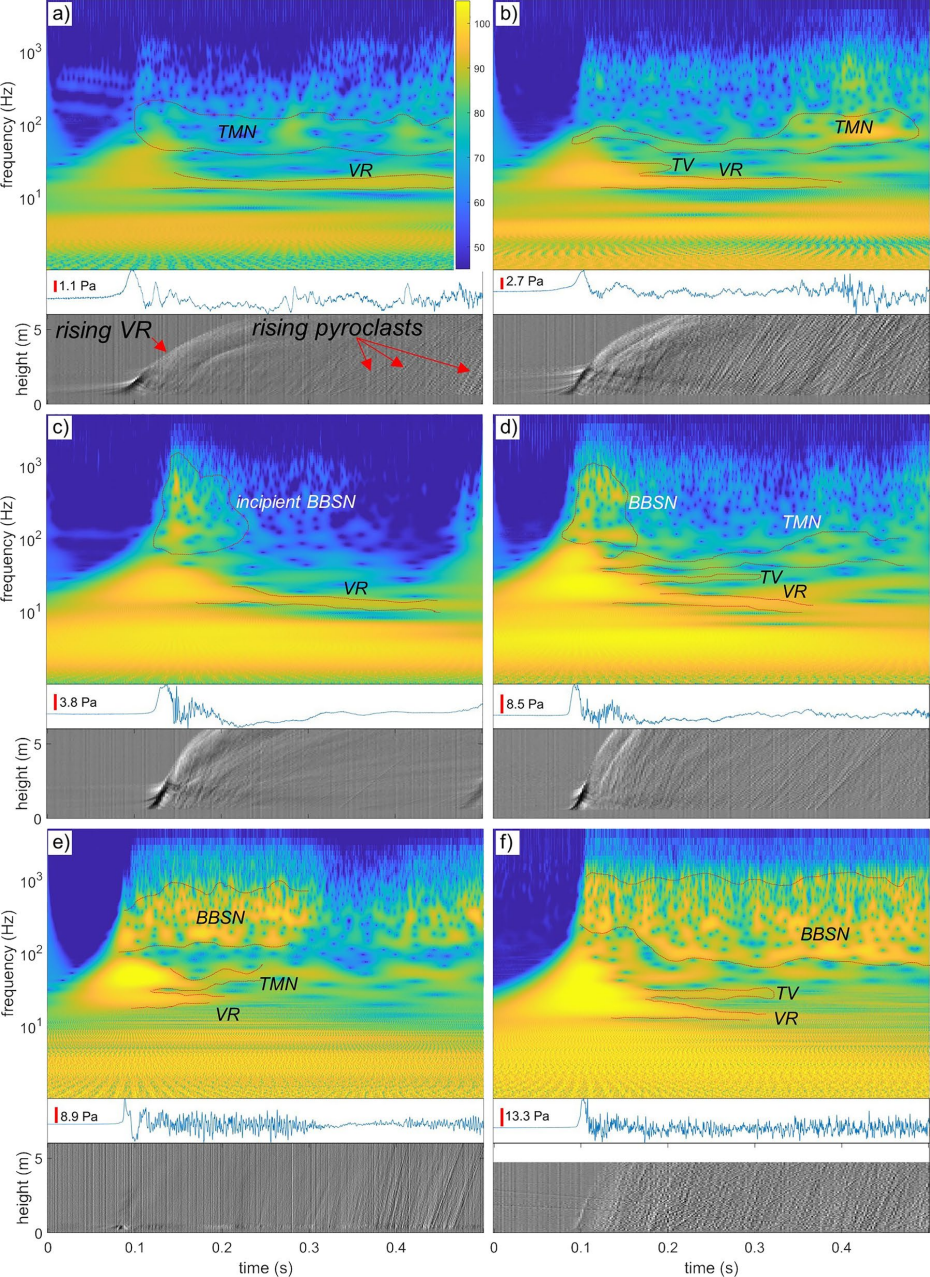
Acoustic and visual measurements of volcanic vortex rings. For each vortex ring, three panels are shown: (1) the wavelet diagram of the acoustic time‐frequency evolution (upper panel; in color scale, the sound pressure level in decibel at the microphone); (2) the acoustic waveform (middle panel, in red the scale of pressure amplitude); and (3) the rise diagram, that is, the time evolution of the mean gray level variations along the central swath of the high‐speed video (see Figure 1), shifted in time to correct for the travel time of the acoustic waves from the vent to the microphone (lower panel). Rising vortex rings produce the broad white to black stripe (large gray tone variations) at the beginning of the event, simultaneous with the first acoustic peak. Rising pyroclasts produce the narrow diagonal lines (smaller gray tone variations), steeper stripes mark higher rise velocity. Vertical lines are noise from the high‐speed camera sensor. Red dotted lines outline acoustic features mentioned in the text: vortex ring (*VR*); trailing vortex (*TV*); turbulent mixing noise (*TMN*); and broadband shock noise (*BBSN*). Panels (a–f) illustrate events with increasing peak amplitude. The same analysis for all cases is in Figures S1–S32).

The formation of VVRs is accompanied by the emission of characteristic acoustic signals. When corrected for the line‐of‐sight travel time of acoustic waves from the vent to the microphone, the acoustic emission appears to be simultaneous (within error of the calculation of sound velocity in air, 348 m/s at 26°C, RH = 49%, and 90.7 kPa, the emergence of VVRs on video, and the onset of the acoustic signal, ±0.02 s ca.) with VVRs formation at the vent. The acoustic signal starts with a well‐defined, asymmetric positive peak whose amplitude rise over time following closely the emergence and rise of the associated VVR (Figures 2 and S2–S33). This peak is interpreted as the compression wave generated by the sudden release of pressure from a gas reservoir inside the conduit and through the vent (e.g., Kieffer & Sturtevant, 1984; Taddeucci et al., 2014). This peak is a jump in pressure that excites all frequencies at the start of the event and produces in the wavelet analysis diagram a characteristic “Mt. Fuji” cone shape whose peak coincides with the arrival of the compression wave at the microphone (Figure 2). The compression wave is followed by a complex signal with frequencies mainly in the 10–1,000 Hz frequency range, often with multiple, distinct frequencies. The characteristic acoustic signature of vortex rings is present in the wavelet analysis of all recordings as a ∼20 Hz frequency component that usually lasts for more than 0.5 s. This component is generated by the stable VVR structure, and displays slight and gradual changes in frequency caused by changes in the velocity of the VVR. Similar but shorter components at frequencies around 40–60 Hz are attributed to individual, large trailing vortices (Figure 1). With increasing vigor and duration of the explosions, marked by higher amplitude of the first peak, the power content above 80 Hz increases and displays more complex features up to 1 kHz in frequency. This higher‐frequency sound increases in amplitude and duration when a relatively sustained jet follows the VVRs (Figures 2 and S1–S32).

**Figure 3:**
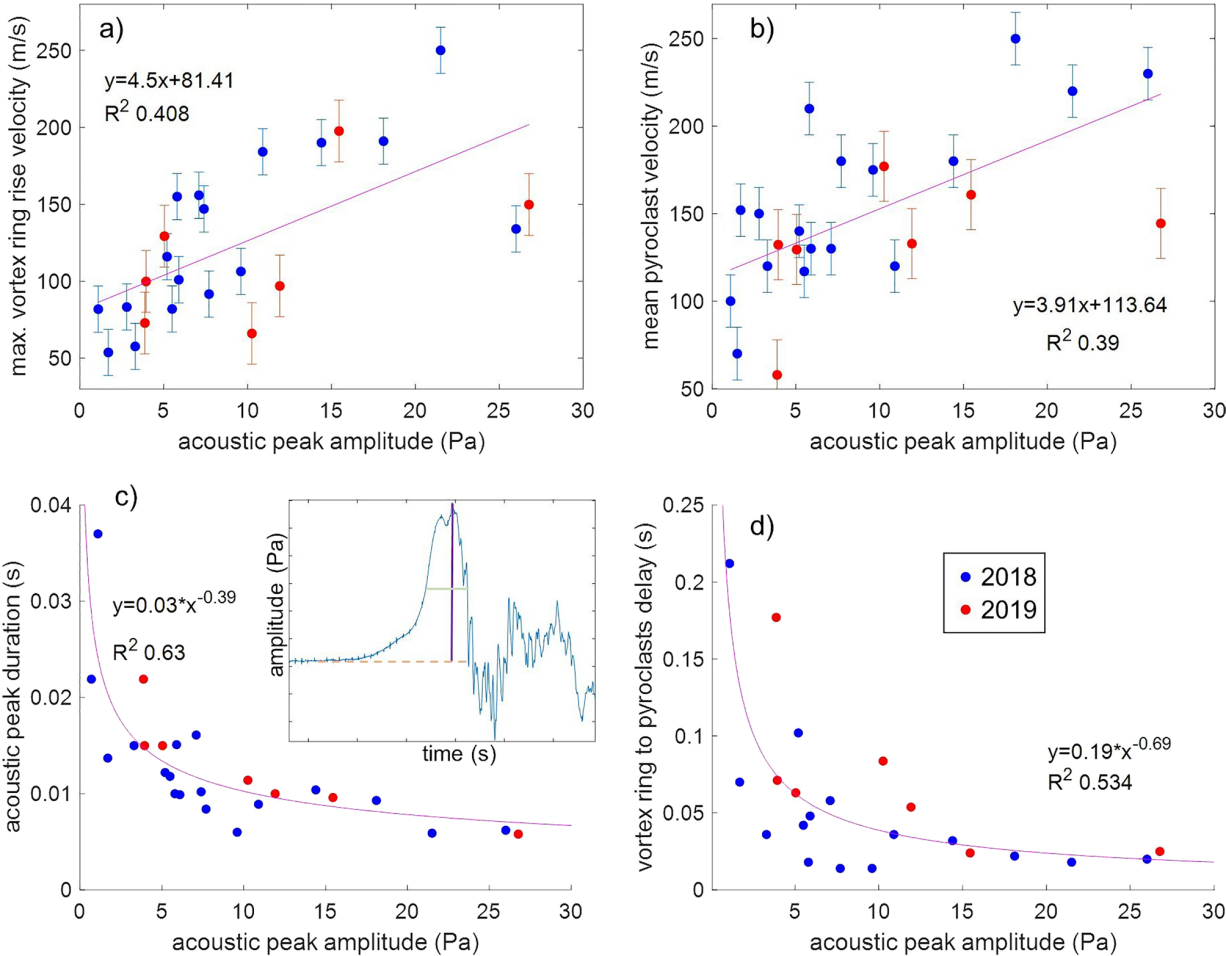
Relationships between the amplitude of the first acoustic peak and: (a) the maximum ascent velocity of vortex rings; (b) the mean ejection velocity of pyroclasts in the first 0.1 s of the jet; (c) the duration of the first acoustic peak (in the inset, the acoustic peak amplitude is shown in purple against the baseline in dashed tan and the half‐duration, in light green); and (d) the duration of the time interval between the appearance of the vortex ring and of the pyroclasts at the vent. In purple, the best‐fit to all data points.

Stronger explosions result in higher amplitudes of: (i) the first acoustic peak, (ii) the maximum rise velocity of the associated VVR, and (iii) the mean velocity of the pyroclasts ejected within the first 0.1 s from the formation of the VVR, all three parameters being weakly correlated positively (Figure 3). The amplitude of the first acoustic peak is instead nonlinearly negatively correlated with its duration and with the time interval between the emergence of the VVR and the ejection of pyroclasts from the vent (Figure 3).

**Figure 4:**
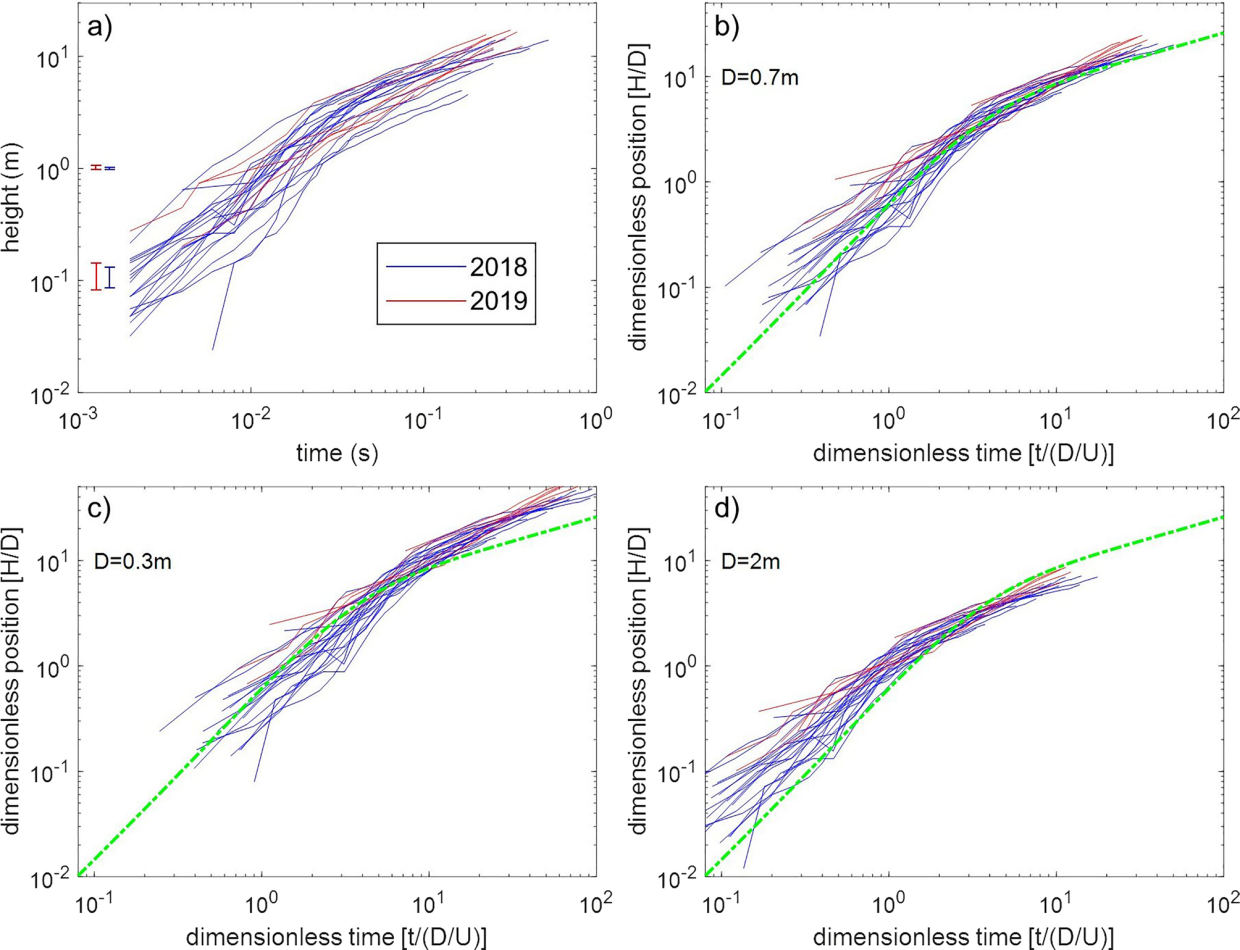
Rise of volcanic vortex rings over time. (a) Vertical position of the head of the vortex rings above the vent as a function of time (aligned in time using the first appearance of the vortex ring at the vent). *y*‐axis error is ±2 pixels. (b) Dimensionless position of the head of the vortex rings as a function of dimensionless time. (c and d) the results of the same fitting for a vent diameter of 0.3 and 2.0 m, respectively. The green line is the model for the dimensionless axial rise of vortex rings by Peña Fernández and Sesterhenn (2019). In the dimensionless units, *t* is time, *H* is height, *D* is vent diameter, and *U* is the mean velocity of each vortex ring. *D* = 0.7 m provides the best fit of the measurements to the model.

Image analysis reveals that the rising of all analyzed VVRs over time broadly follows two distinct power law trends: a steeper one during the first about 0.05 s, and a gentler one afterward (Figure 4). By using their own direct numerical simulation and literature experimental work (not including VVRs), Peña Fernández and Sesterhenn (2019) assembled a comprehensive overview of axial and radial dynamics of vortex rings that cover a broad variety of physical conditions. They used this database to define a general model for the axial rise of vortex rings, scaling the axial position of vortex rings against vent diameter, and scaling time against vent diameter divided by the jet velocity. Applying the general model of Peña Fernández and Sesterhenn (2019) to our VVRs requires two unknown data: vent diameter and jet velocity. By varying systematically vent diameter and velocity we found that the combination that best matches (by least squares optimization) the theoretical curve is setting vent diameter equal to 0.75 m and velocity equal to the mean velocity of the VVRs (Figure 4). The fit is in general good, with only a minor upward deviation of the 2019 data in the later stage of the VVRs rise.

## 4 Acoustic Features of Volcanic Vortex Rings and Supersonic Jet Flow

We interpret our acoustic signals in light of recent shock‐tube experiments specifically designed to mimic unsteady volcanic eruptions (Peña Fernández et al., 2020). The characteristic frequency of vortex ring noise depends on vortex ring rise velocity, size, and vent diameter (Kopiev, 2000; Ran & Colonius, 2009). This frequency could be used to measure vent diameter, but only by measuring the outer and inner diameter of VVRs in addition to their rise velocity. However, since this frequency is mostly unrelated to jet overpressure and duration (Peña Fernández et al., 2020), we remark that the observed stability of this frequency in our data set suggests a stable vent diameter over the different explosions we observed, in agreement with UAV observations. As the amplitude of the first acoustic peak, proxy for explosion power, increases, higher‐frequency acoustic components in a broad band from ∼80 to 1,000 Hz gradually appear and intensify. These components are interpreted as the jet noise already recorded in jet‐forming explosions at Stromboli (Taddeucci et al., 2014). This includes turbulent mixing noise (TMN), at lower frequency and lower jet velocity, and broadband shock noise (BBSN), having a generally broader and higher‐frequency range that, as the jet becomes increasingly supersonic, overlaps and dominates in amplitude over the TMN. The BBSN frequency is linked mainly to vent diameter and the Mach number inside the jet (e.g., Matoza et al., 2013; Peña Fernández et al., 2020; Tam, 1995). By using the observed vent diameter (Figure 1) and the observed range of BBSN frequency (Figure 2), Equation 3 of Peña Fernández et al. (2020), provided below, can be used to estimate the maximum Mach number within the measured volcanic jets:

(1) $$f_{BBSN}=\frac{\sigma_{1}}{\pi \sqrt{M_{j}^{2}-1}}\frac{c_{j}}{D_{j}},$$

where *f*_*BBSN*_ is the peak BBSN frequency, *σ*_1_ is the first root of the zero‐order Bessel function, *c*_*j*_, *M*_*j*_, and *D*_*j*_ are the speed of sound, the Mach number, and the jet diameter at fully expanded conditions, respectively, and are calculated after Peña Fernández et al. (2020). Given the large uncertainties in determining the peak BBSN frequency linked to the presence of other jet noise components (Matoza et al., 2013; Tam, 1995) and the natural environment of the recording, we estimate a maximum Mach number in the jets in between 1.5 and 2.

This method opens the possibility of using frequency shifts in volcano acoustic measurements to infer changes at subsecond scale in jet exit velocity (and hence mass eruption rate) at the vent during explosive eruptions.

In analogy with shock‐tube experiments, VVRs are generated as soon as the shock wave from the pressure release (i.e., gas pocket burst) in the conduit reaches the vent and jet emission starts (Salvatore et al., 2020). At the same time, the compression wave leaves the vent, as supported by the lack of any discernible delay between VVR appearance and compression wave emission. The delay between the appearance of VVRs and pyroclasts at the vent (Figure 3), and its inverse, non‐linear relationship with pressure peak amplitude, is thus analogous to the delay between the time of acoustic emission and that of pyroclasts ejection that has been used to estimate the depth of explosions within the conduit (Delle Donne & Ripepe, 2012). This depth estimate relied on the assumption that acoustic waves were generated at depth by the burst and then traveled to the vent at the velocity of sound within the conduit. However, for specific combinations of initial pressure and conduit size, we suggest that the acoustic waves leaving the vent travel in the conduit as a shock wave (Salvatore et al., 2020) and that the time delay reflects the different travel time of pyroclasts and shock waves, not acoustic waves, within the conduit. The synchronous emission of VVR and compression wave opens the possibility of measuring the acoustic‐to‐particle emission delay by visual means, without acoustic measurement and thus independent of the travel time of acoustic waves from the vent to the microphone. The amplitude of the first acoustic peak correlates weakly positive with both VVRs and pyroclasts rise velocity, but not along the power law previously observed at Stromboli (Delle Donne & Ripepe, 2012). This discrepancy could be due to differences in: (1) the style of the recorded explosions (cf. Leduc et al., 2015) and of the corresponding pressure and frequency of the first acoustic waves, and (2) the methods used to measure exit velocity (thermal versus visible videos at 50 versus 500 fps) and acoustic signals (infrasound vs. broadband microphones). Conversely, the first peak amplitude correlates inversely with its duration. These correlations suggest that peak pressure is related to both the initial pressure difference and the volume of gas that drove the explosion and controlled the VVRs and pyroclast exit velocity (Peña Fernández et al., 2020; Salvatore et al., 2020).

## 5 Axial Translation of Volcanic Vortex Rings and Vent Diameter Retrieval

The axial motion of vortex rings follows two trends, an initial one related to the formation stage, in which position is proportional to time *τ* ^1.5^, and a following one related to the translation stage where position is proportional to time *τ* ^0.5^ (Didden, 1979; Peña Fernández & Sesterhenn, 2019; Witze, 1980). The transition between these trends occurs when the dimensionless time is between 1 and 10. The VVRs that we measured follows well the trend predicted by Peña Fernández and Sesterhenn (2019) (Figure 4). Not knowing the exit velocity of the gas jet at the vent, we normalized time by using the average rise velocity of the VVRs in the first 10 meters above the vent as a characteristic velocity. An attempt to normalize time using the maximum VVR rise velocity yielded similar results but a larger error. Note that, while vent diameter enters in both dimensionless position and time, velocity is used only for dimensionless time, so that only one couple of diameter‐velocity values can adequately fit the data to the model. The use of the average velocity right above the vent, which can be measured from video, allows an independent estimate of vent diameter at 0.7 m, matching well the one inferred from direct observation by UAV and observation of the diameter of the jet at the vent (Figure 1), supporting the use of VVRs measurements to infer vent diameter in jet‐forming explosions. For our study cases, VVRs rise velocity seems to be unaffected by the funnel shape of the vent and its large outer rim (∼3.5 m) (Figure 4), suggesting a relatively low degree of jet overpressure with respect to the external environment (e.g., Saad, 1985).

Factors that can hamper the volcanological application of this fluid‐dynamic model include the lack of a parameterization for non‐circular vents and the large variability of volcanic jets. The 2019 explosions produced VVRs that tend to deviate toward faster rise at late dimensionless times compared to the model and the 2018 cases (Figure 4), possibly due to the sustained (at the observational time scale) nature of the jets. Multiple VVRs generated at short intervals within a single explosion may also interfere with one another to deviate from the empirical model, which is based on simpler numerical and experimental cases. Another difference between VVRs and other vortex rings is that the thermal content of volcanic gases and ash particles in the former induces a buoyancy force that contributes to their translation, and all transitions from momentum‐dominated to buoyancy‐dominated VVRs will exists. At Stromboli, Patrick (2007) used an exit velocity threshold of 15 m/s to discriminate gas‐thrust from buoyant plumes, both plume types featuring VVRs, but the former rising according to time *τ* ^0.62^ and the latter to time *τ* ^0.81^. However, the same rising trends and plume morphologies of Patrick (2007) were replicated in neutral buoyancy experiments (Chojnicki et al., 2014, 2015), questioning the role of buoyancy on the motion of these VVRs. The time scale of our observations (<1 s), the range of exit velocities, and the measured time proportionalities all exclude any buoyancy contribution in the motion of our study cases. However, any attempt to interpret VVR measurements at the longer time scales, as in most Vulcanian eruptions (Tournigand et al., 2017), should consider this factor.

## 6 Conclusions

Estimating eruption source parameters is crucial for eruption modeling and volcanic hazard assessment, and the hunt for new methods to retrieve them is a prime task of the geophysical community. Here, we used a new, high‐sampling rate (500 fps video and 10 kHz audio) parameterization of the acoustic and translation features of volcanic vortex rings to retrieve two key eruption source parameters: the Mach number of the eruption jet and the diameter of the eruptive vent. Both parameters have implications for the estimate of mass eruption rate, but also the assessment of the source depth of the explosions and the interpretation of the eruption acoustic signals. The methodologies that we propose are a first step toward the detection of the variations of these eruption parameters at a temporal scale of seconds or less.

## Conflict of Interest

The authors declare no conflicts of interest relevant to this study.

## Supporting information

Supporting Information S1

## Data Availability

Data sets for this research are available in Mendeley Data, v1, http://dx.doi.org/10.17632/yhbzrsbyp5.2.
