# Supplementary material for: Volcanic Vortex Rings: Axial Dynamics, Acoustic Features, and Their Link to Vent Diameter and Supersonic Jet Flow
Source: Geophys Res Lett. 2021 Jul 28;48(15):e2021GL092899. doi: 10.1029/2021GL092899 (PMC8365711; doi:10.1029/2021GL092899)
Supplement: Supplementary file 1 — Supporting Information S1 [file GRL-48-e2021GL092899-s001.pdf]

AGU PUBLICATIONS

Geophysical Research Letters

Supporting Information for

Volcanic Vortex Rings: Axial Dynamics, Acoustic Features, and their Link to Vent Diameter and Supersonic Jet Flow

J. Taddeucci[1], J.J. Peña Fernández[2], V. Cigala[2], U. Kueppers[2], P. Scarlato[1], E. Del Bello[1], T. Ricci[1], J. Sesterhenn[3], S. Panunzi[1]

[1]Istituto Nazionale di Geofisica e Vulcanologia, Rome, Italy.
[2]Ludwig-Maximilians-Universität München, Munich, Germany.
[3]Universität Bayreuth, Bayreuth, Germany.

Contents of this file

Figures S1 to S33

Introduction

Figure S1 is a comparison between wavelet scalogram and short-time Fourier transform spectrograms of one explosion signal. Figures S2 to S33 are composed of three panels and, like Fig. 2 of the manuscript, depict the wavelet scalogram, acoustic waveform, and rise diagrams for all recorded volcanic vortex rings, determined as in Fig. 2. Figure S2 includes the figure legend.

1) A comparison between wavelet scalogram analysis and Short-Time Fourier Transform (STFT) spectrogram analysis of the signal from event **20280912_1123_1**

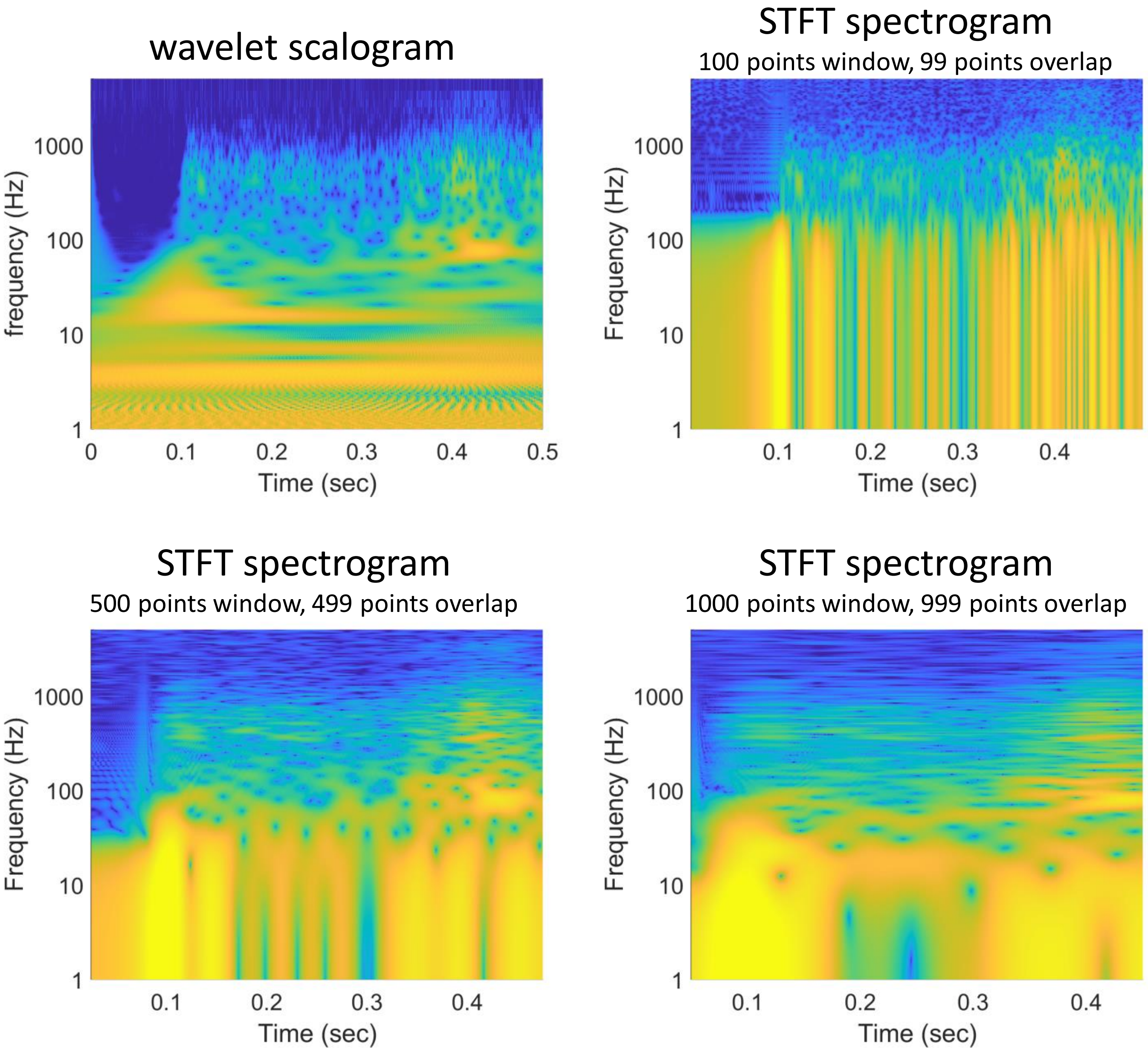

2) Acoustic and video features of the observed volcanic vortex rings.

FIGURE LEGEND

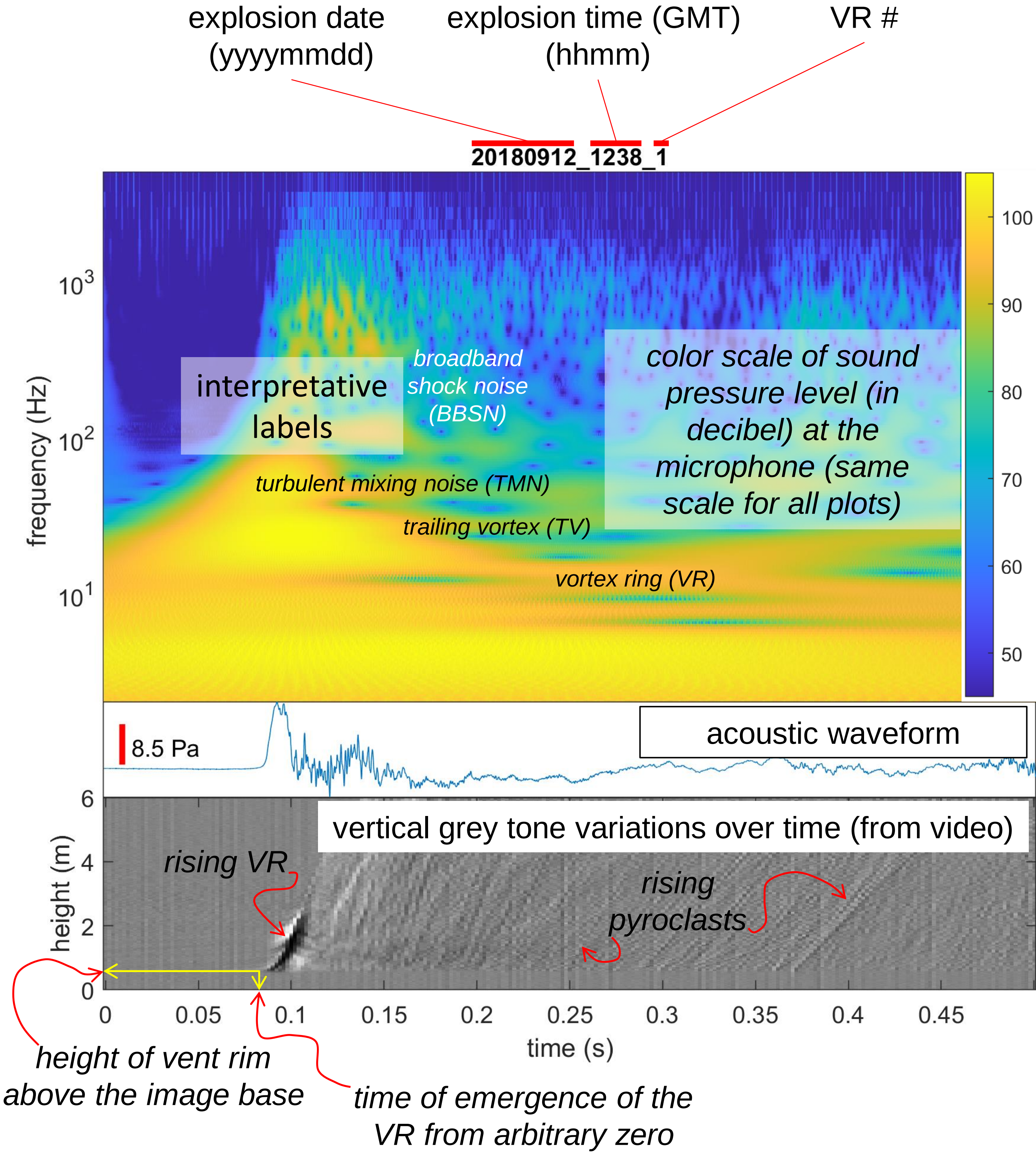

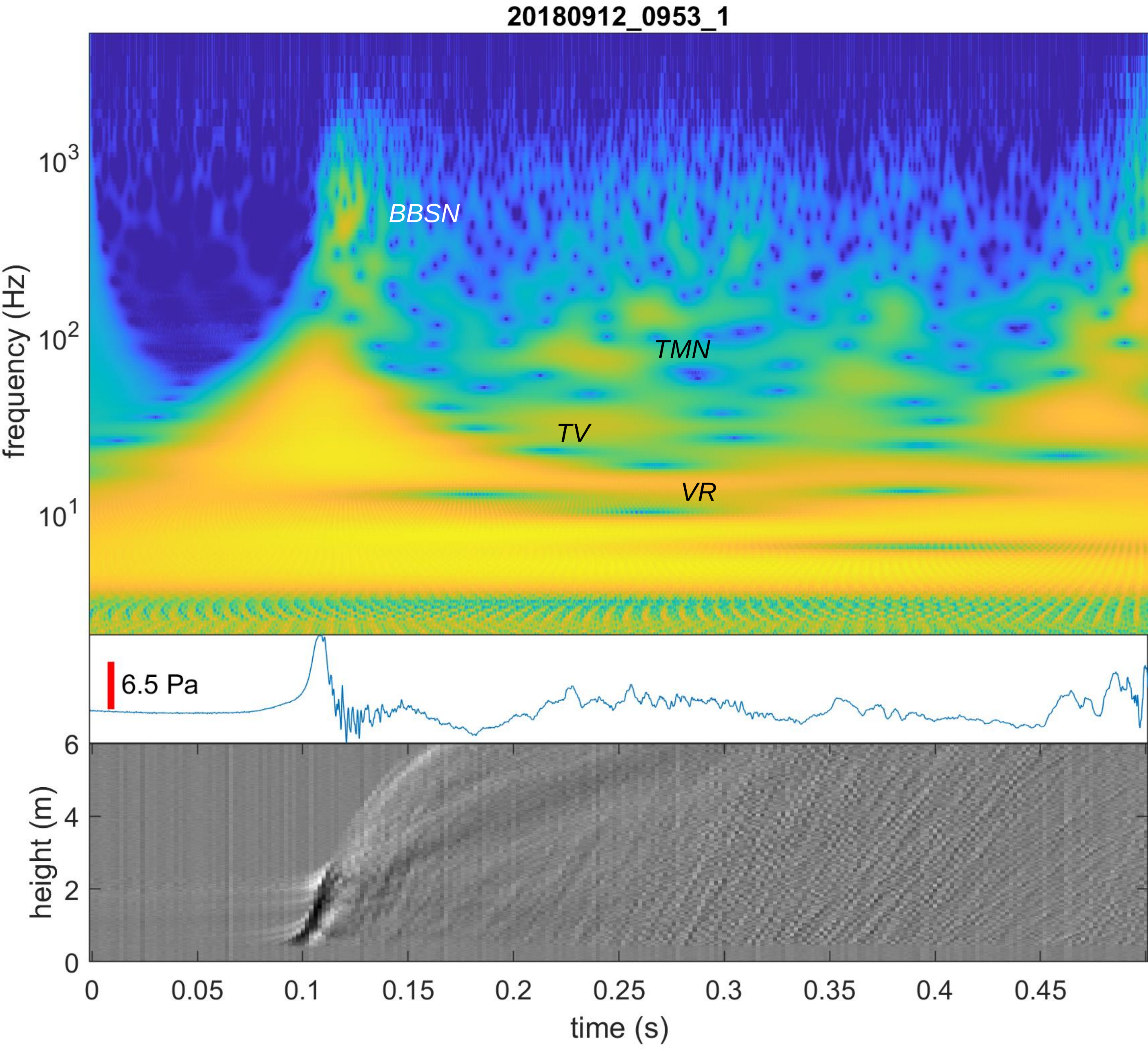

20180912_0953_1
BBSN
TMN
TV
VR
frequency (Hz)
10^3
10^2
10^1
6.5 Pa
height (m)
6
4
2
0
0
0.05
0.1
0.15
0.2
0.25
0.3
0.35
0.4
0.45
time (s)

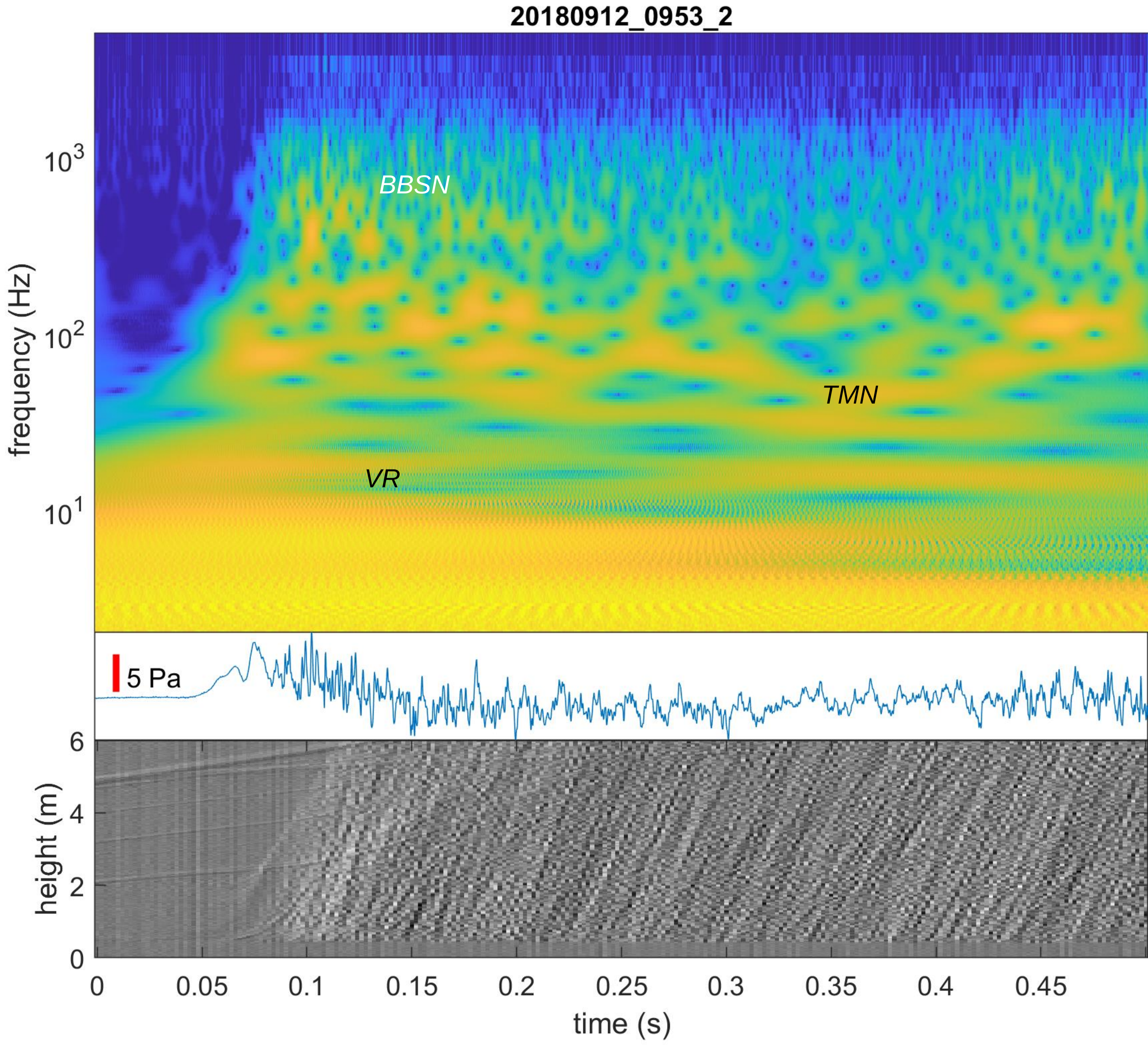

20180912_0953_2
frequency (Hz)
10^3
10^2
10^1
BBSN
TMN
VR
5 Pa
height (m)
6
4
2
0
0.05
0.1
0.15
0.2
0.25
0.3
0.35
0.4
0.45
time (s)

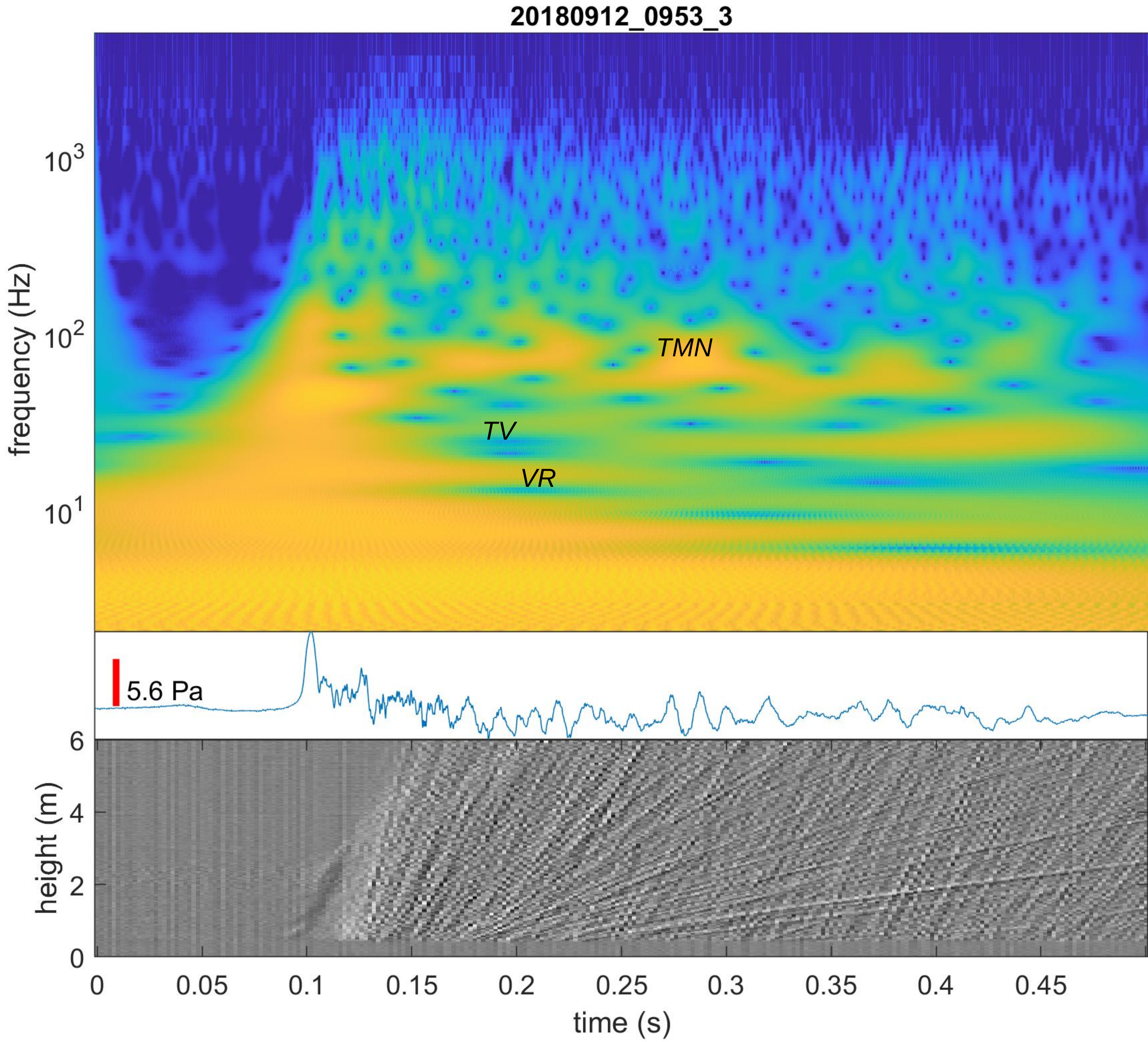
20180912_0953_3
frequency (Hz)
10^3
10^2
10^1
TMN
TV
VR
5.6 Pa
height (m)
6
4
2
0
0
0.05
0.1
0.15
0.2
0.25
0.3
0.35
0.4
0.45
time (s)

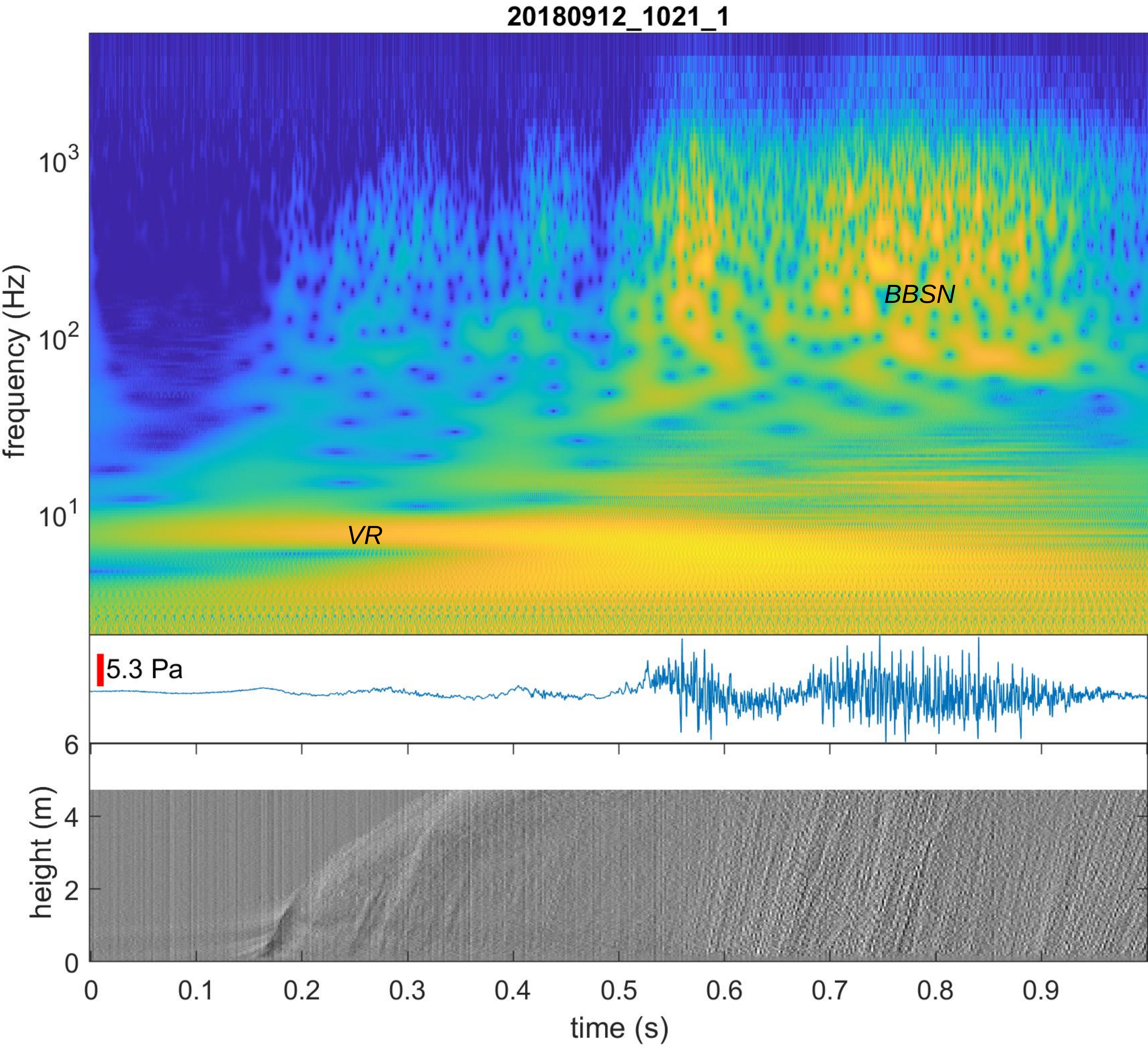
20180912_1021_1
frequency (Hz)
BBSN
VR
5.3 Pa
height (m)
time (s)

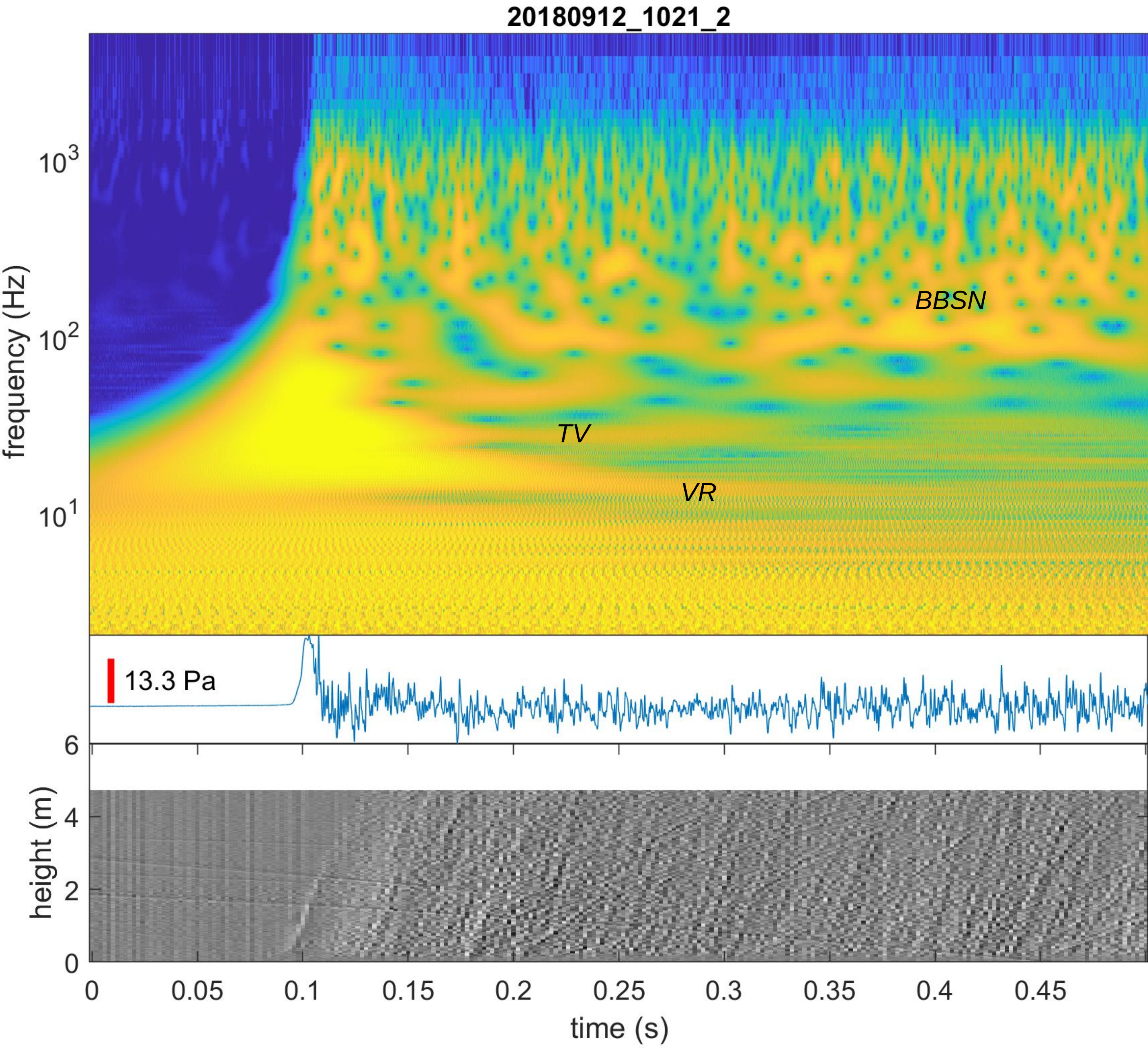

20180912_1021_2
frequency (Hz)
10^3
10^2
10^1
BBSN
TV
VR
13.3 Pa
height (m)
6
4
2
0
0
0.05
0.1
0.15
0.2
0.25
0.3
0.35
0.4
0.45
time (s)

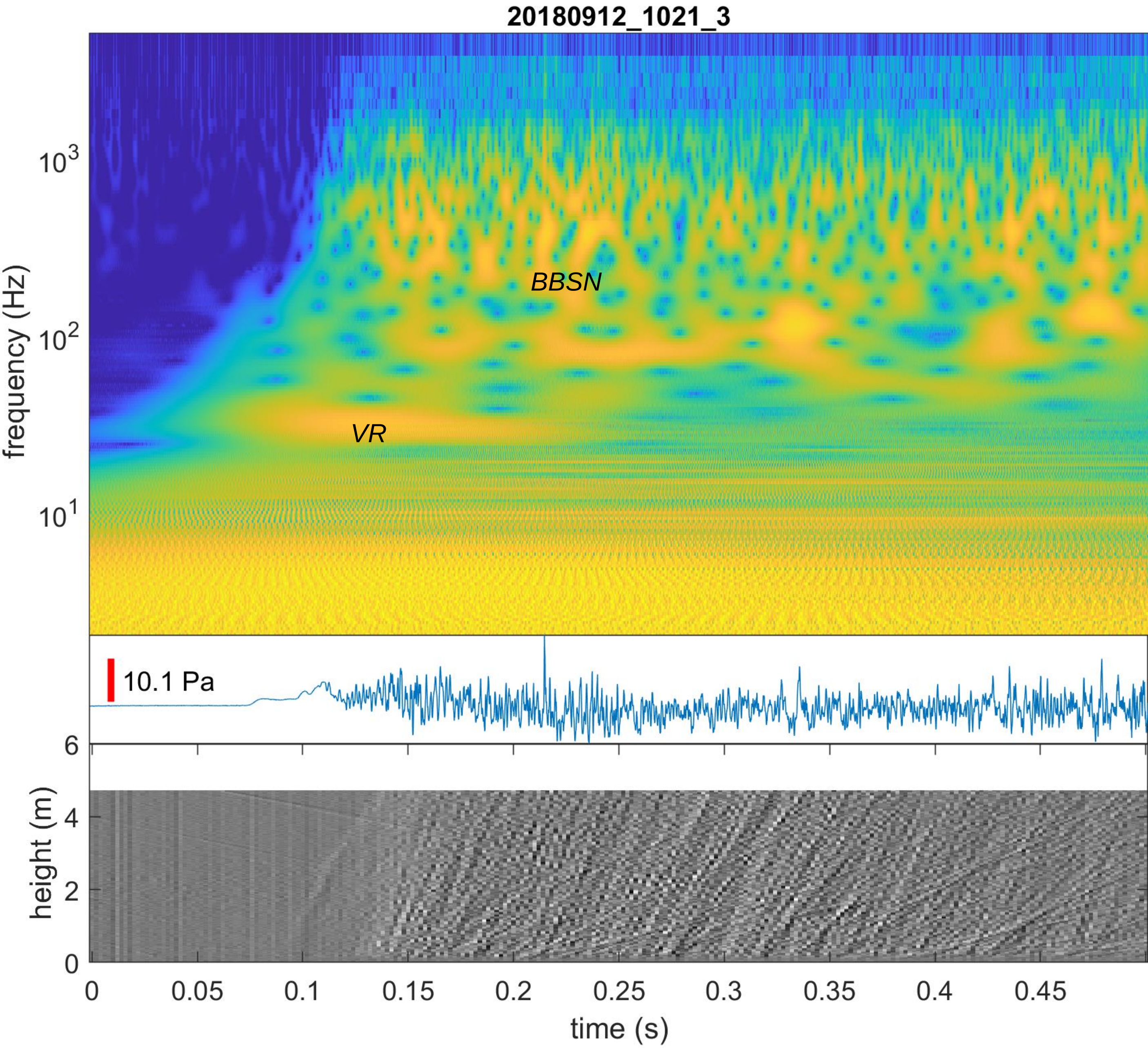

20180912_1021_3
frequency (Hz)
10^3
10^2
10^1
BBSN
VR
10.1 Pa
height (m)
6
4
2
0
0
0.05
0.1
0.15
0.2
0.25
0.3
0.35
0.4
0.45
time (s)

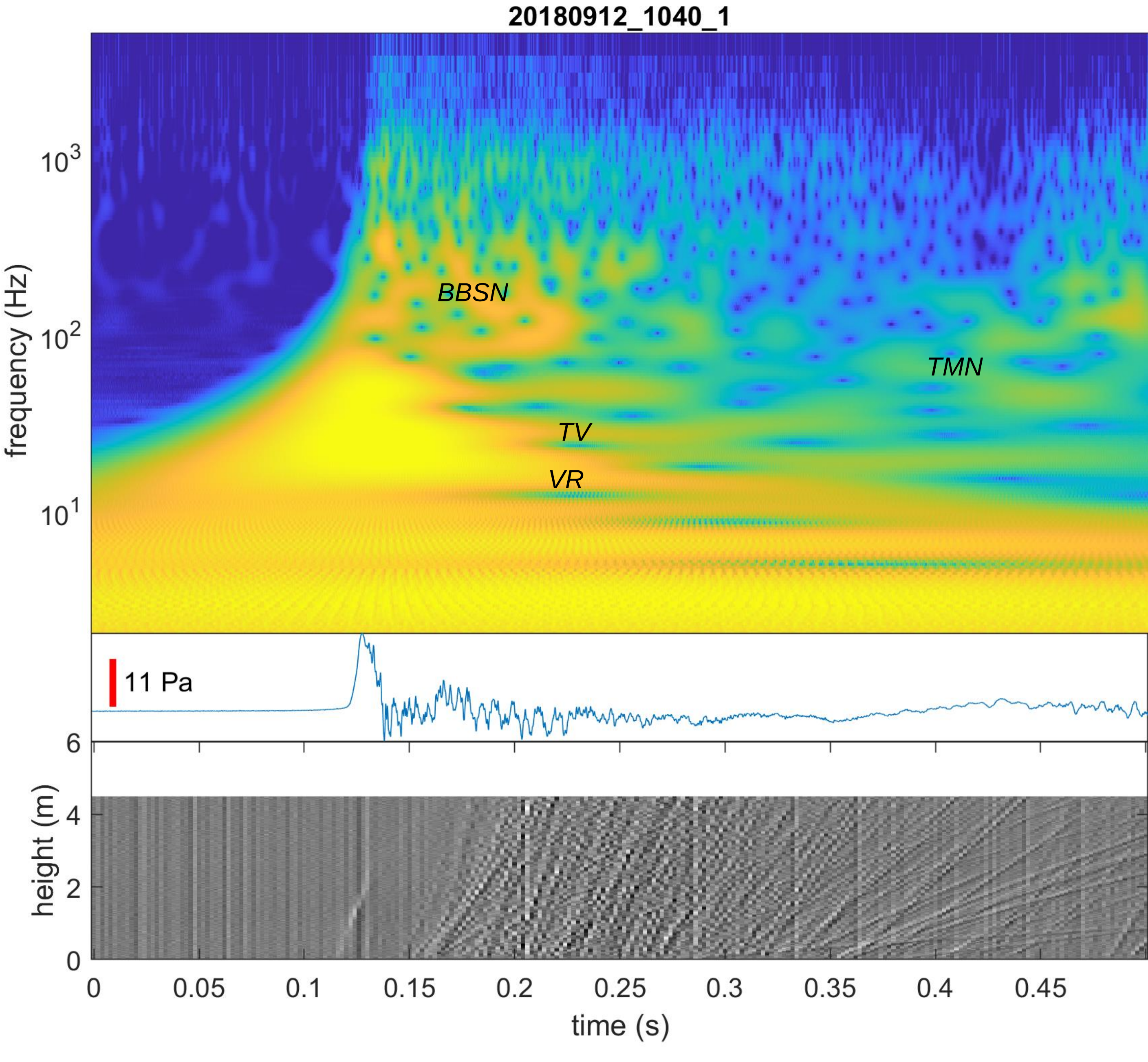
20180912_1040_1
BBSN
TMN
TV
VR
frequency (Hz)
11 Pa
height (m)
time (s)

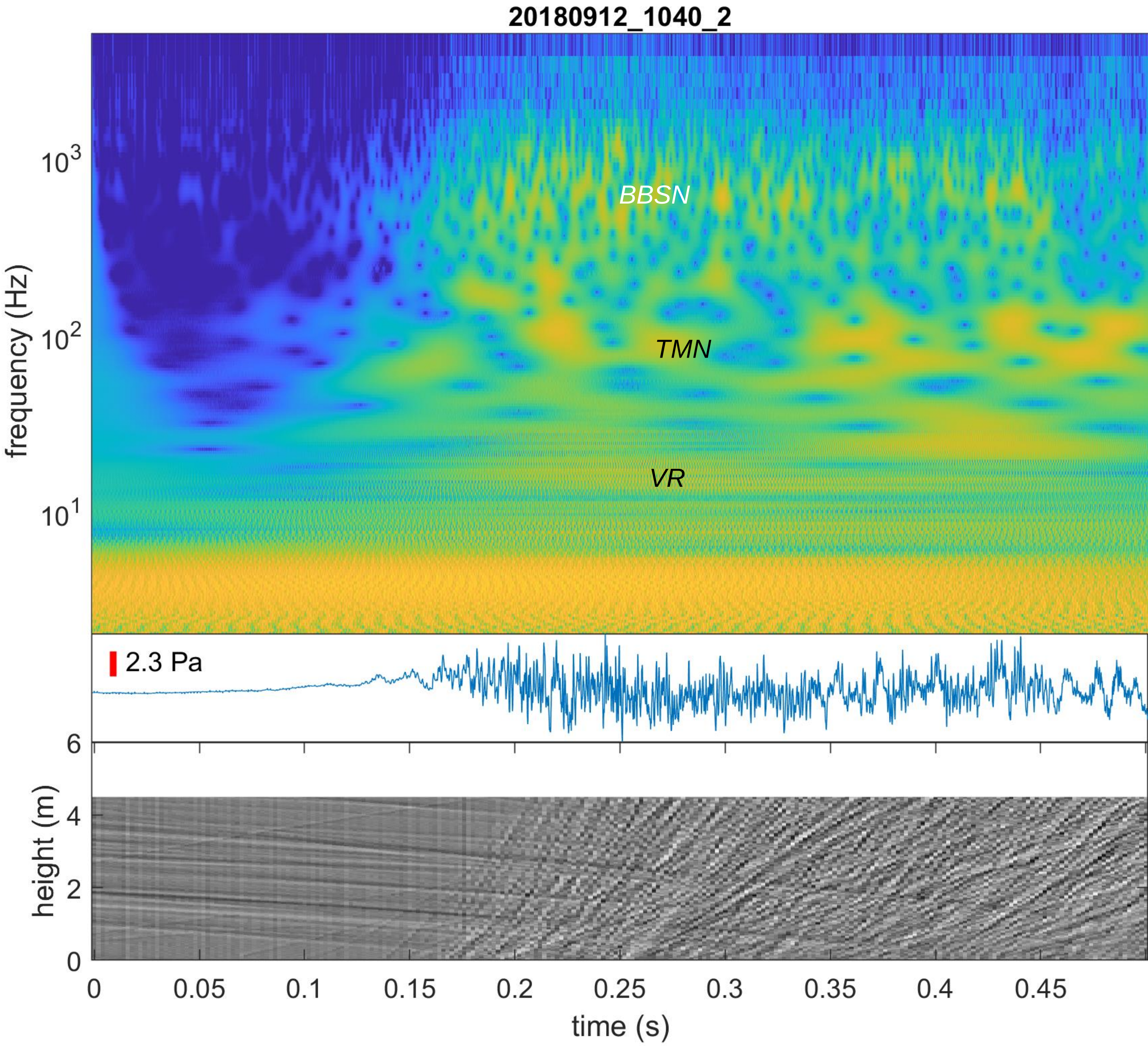

20180912_1040_2
BBSN
TMN
VR
frequency (Hz)
10^3
10^2
10^1
2.3 Pa
height (m)
6
4
2
0
0
0.05
0.1
0.15
0.2
0.25
0.3
0.35
0.4
0.45
time (s)

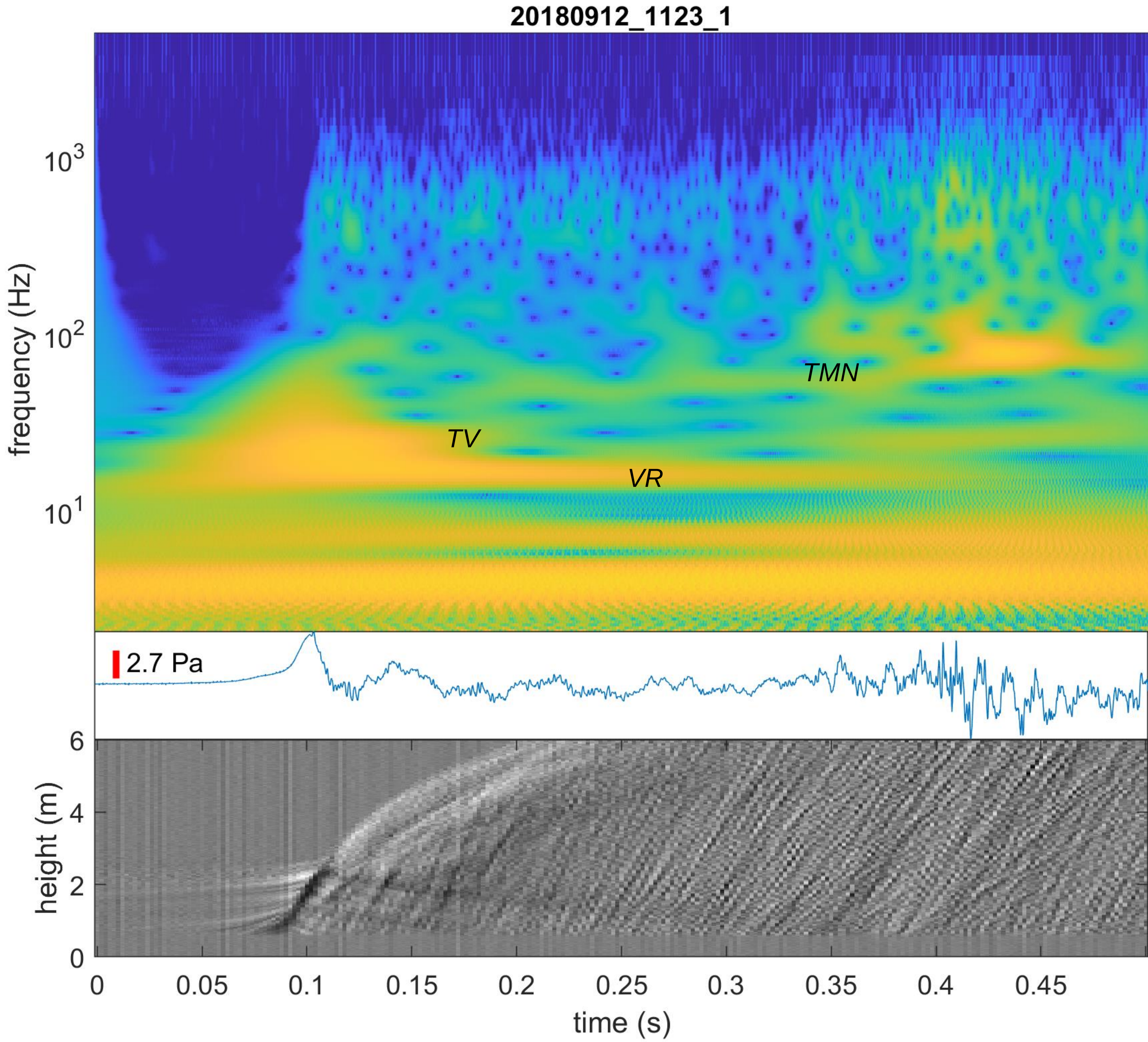

20180912_1123_1
frequency (Hz)
10^3
10^2
10^1
TV
VR
TMN
2.7 Pa
height (m)
6
4
2
0
0.05
0.1
0.15
0.2
0.25
0.3
0.35
0.4
0.45
time (s)

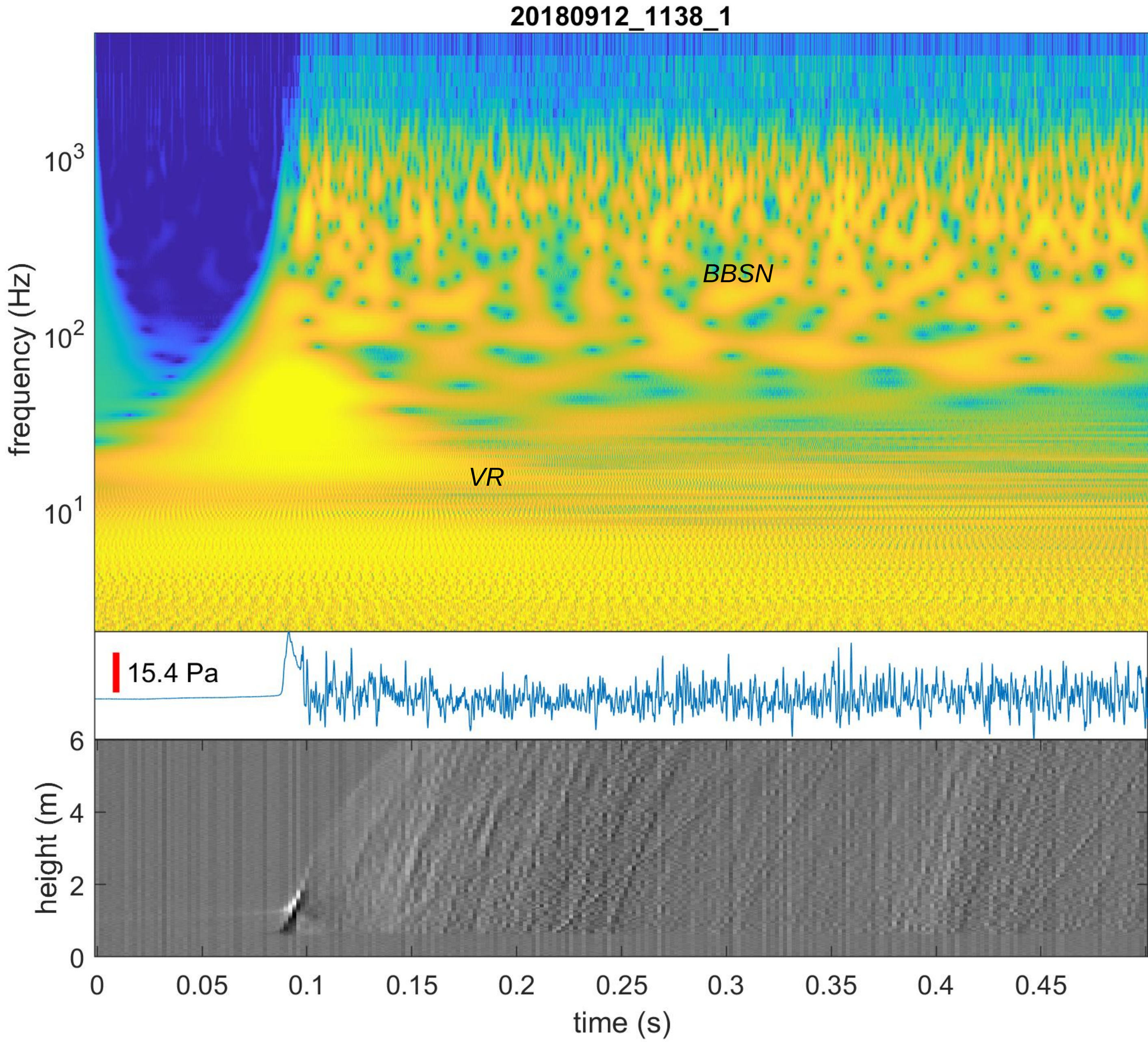

20180912_1138_1
BBSN
VR
frequency (Hz)
15.4 Pa
height (m)
time (s)

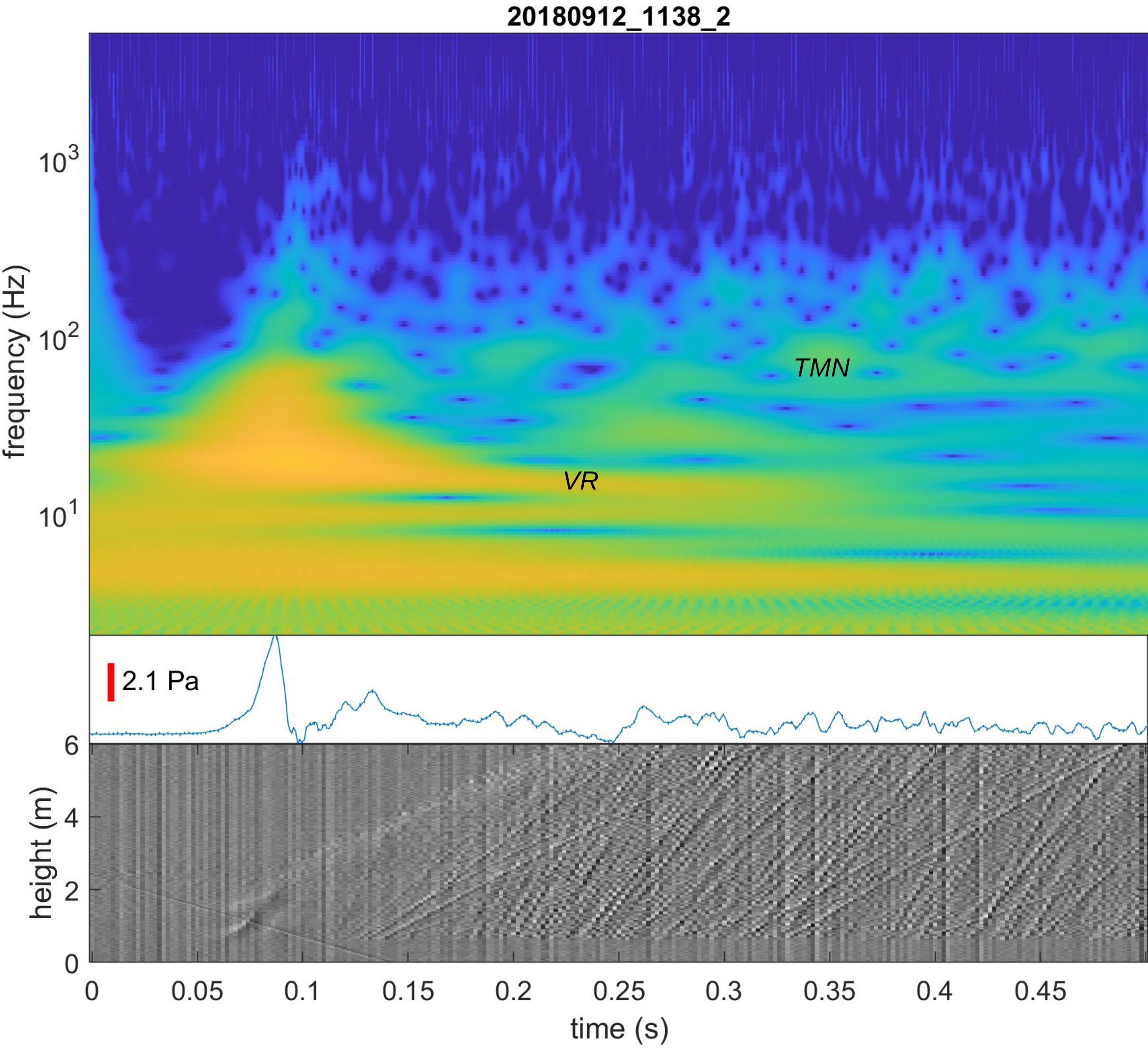

20180912_1138_2
frequency (Hz)
TMN
VR
2.1 Pa
height (m)
time (s)

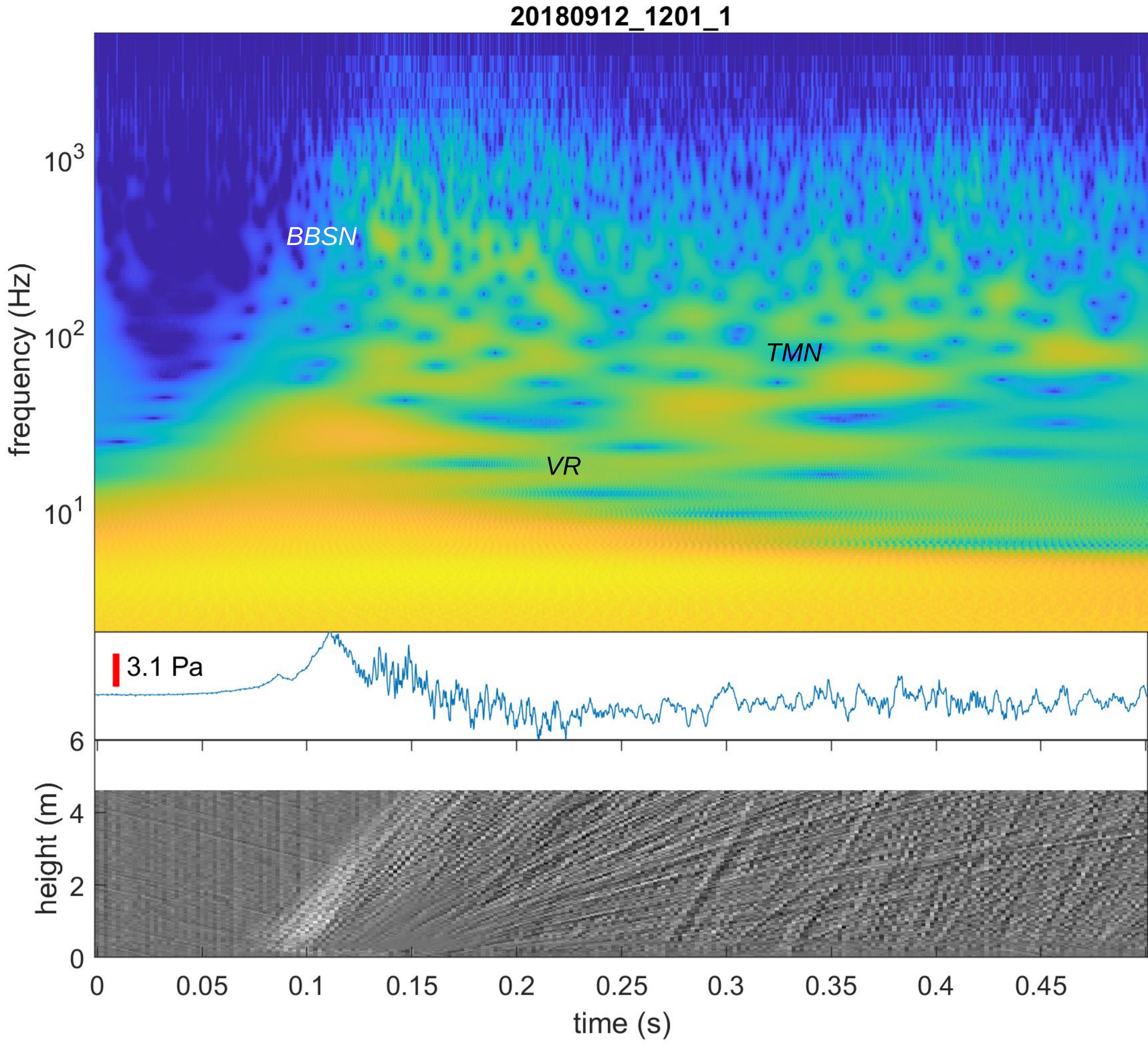

20180912_1201_1
frequency (Hz)
10^3
10^2
10^1
BBSN
TMN
VR
3.1 Pa
height (m)
6
4
2
0
0
0.05
0.1
0.15
0.2
0.25
0.3
0.35
0.4
0.45
time (s)

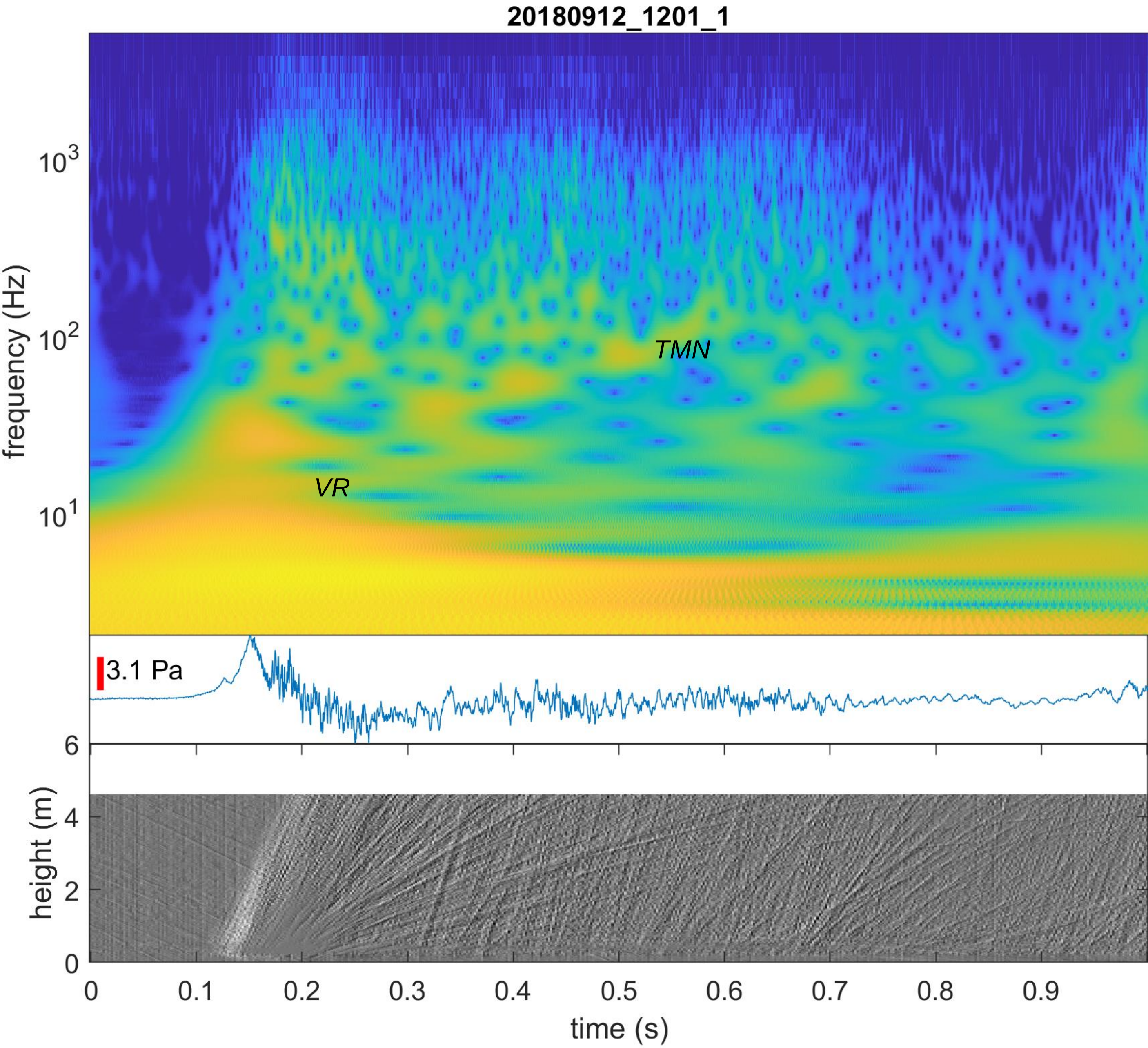
20180912_1201_1
frequency (Hz)
10^3
10^2
10^1
TMN
VR
3.1 Pa
height (m)
6
4
2
0
0
0.1
0.2
0.3
0.4
0.5
0.6
0.7
0.8
0.9
time (s)

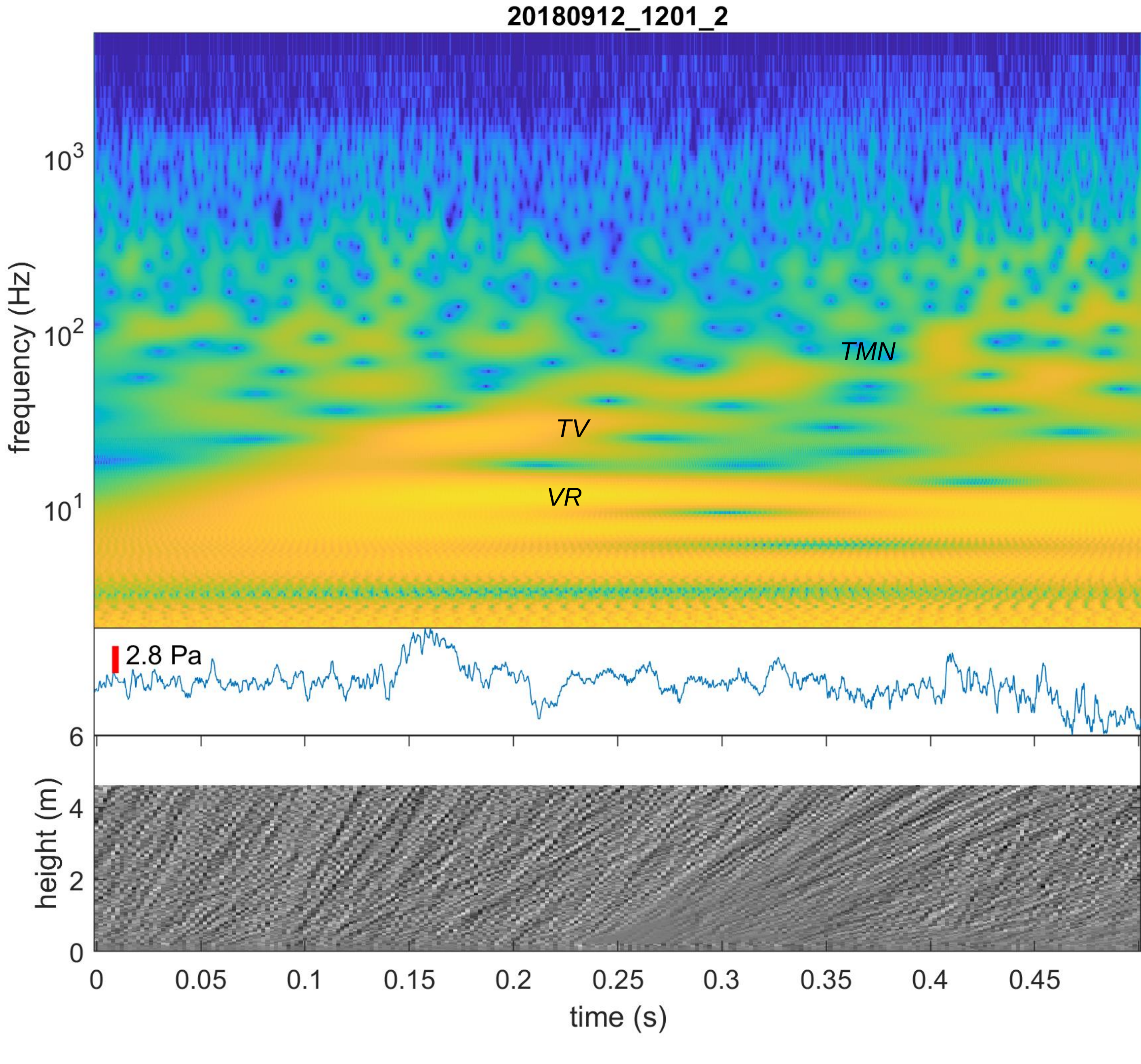
20180912_1201_2
frequency (Hz)
10^3
10^2
10^1
TMN
TV
VR
2.8 Pa
height (m)
6
4
2
0
0
0.05
0.1
0.15
0.2
0.25
0.3
0.35
0.4
0.45
time (s)

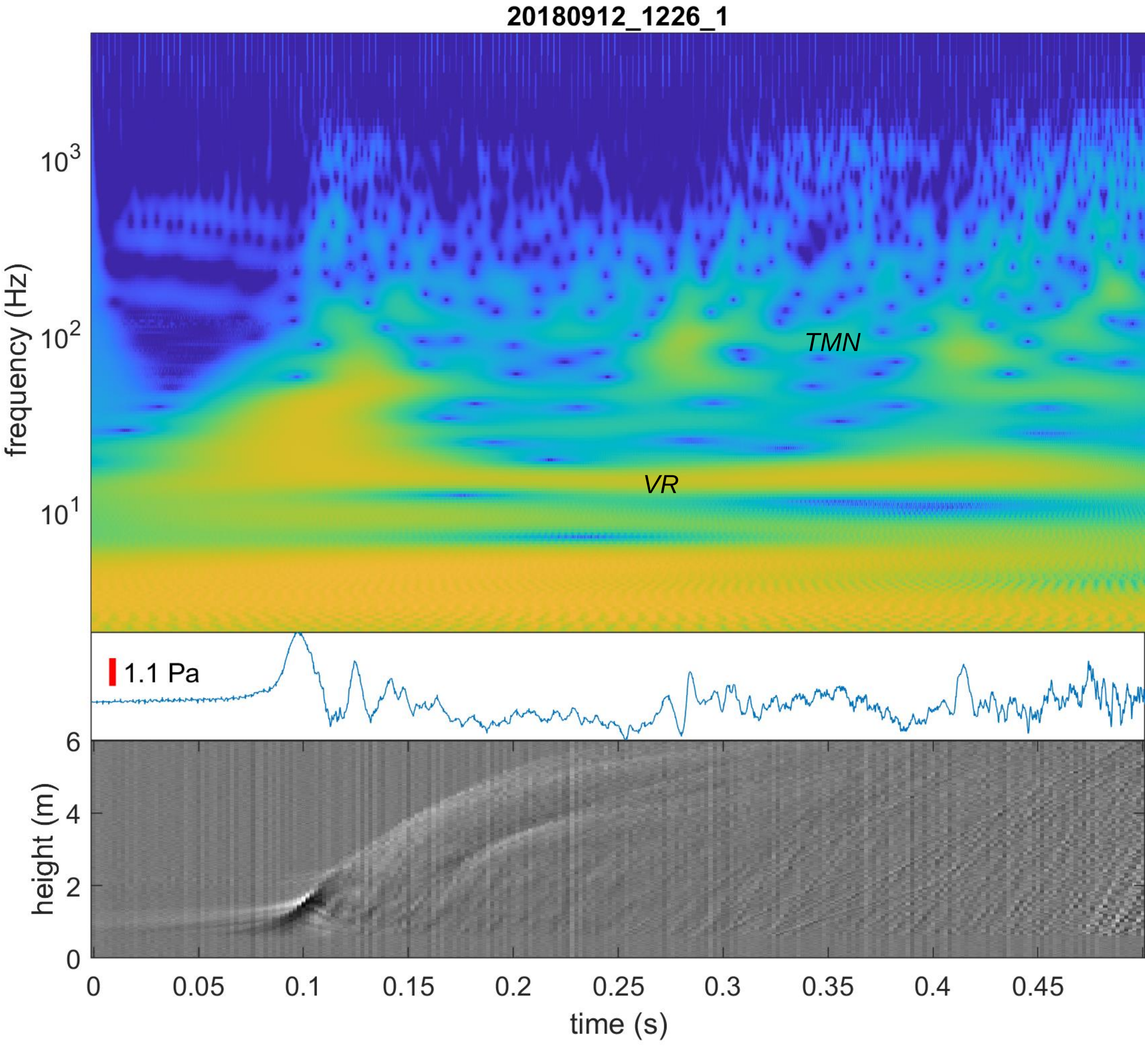

20180912_1226_1
frequency (Hz)
10^3
10^2
10^1
TMN
VR
1.1 Pa
height (m)
6
4
2
0
0
0.05
0.1
0.15
0.2
0.25
0.3
0.35
0.4
0.45
time (s)

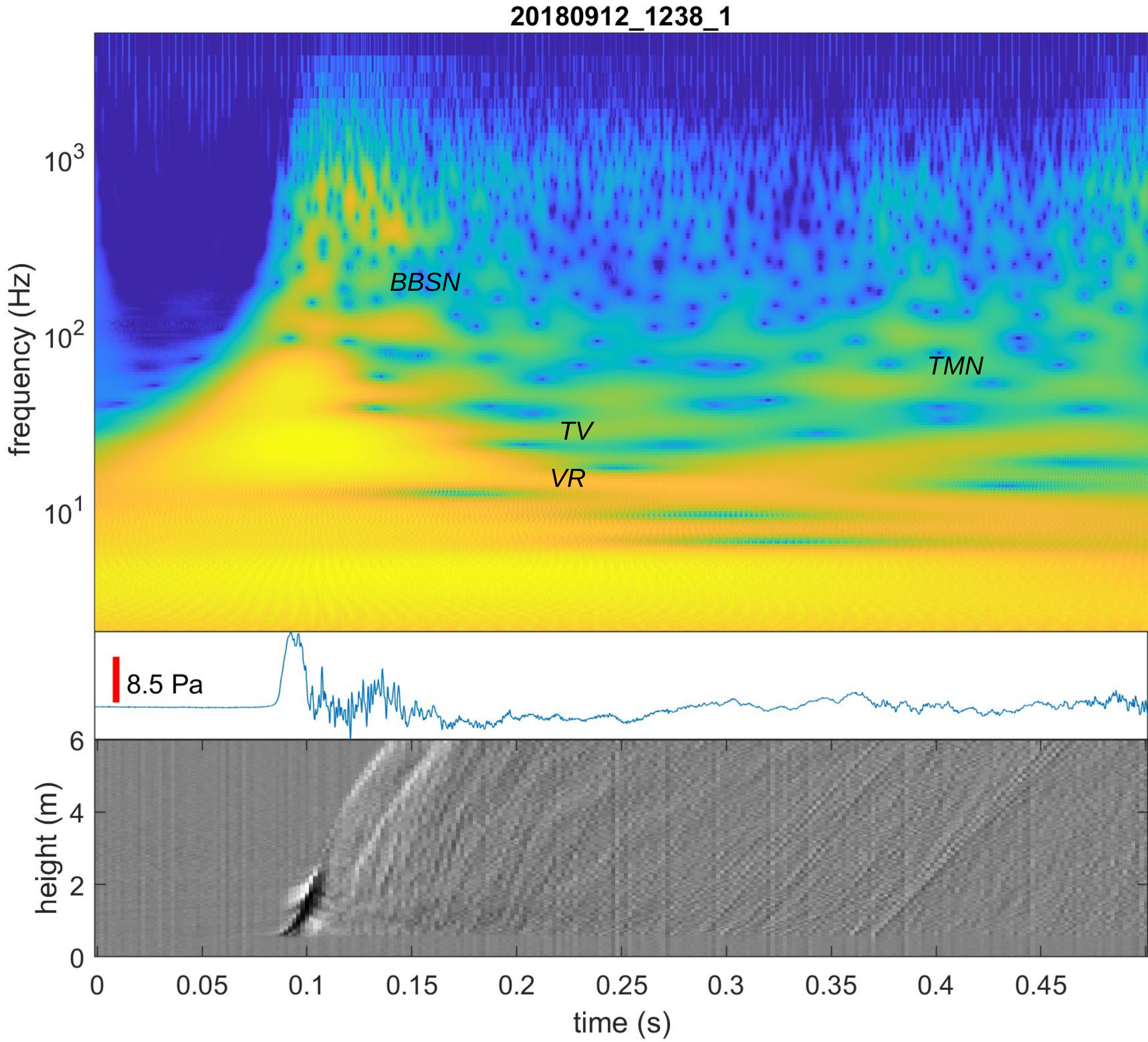

20180912_1238_1
frequency (Hz)
BBSN
TMN
TV
VR
8.5 Pa
height (m)
time (s)

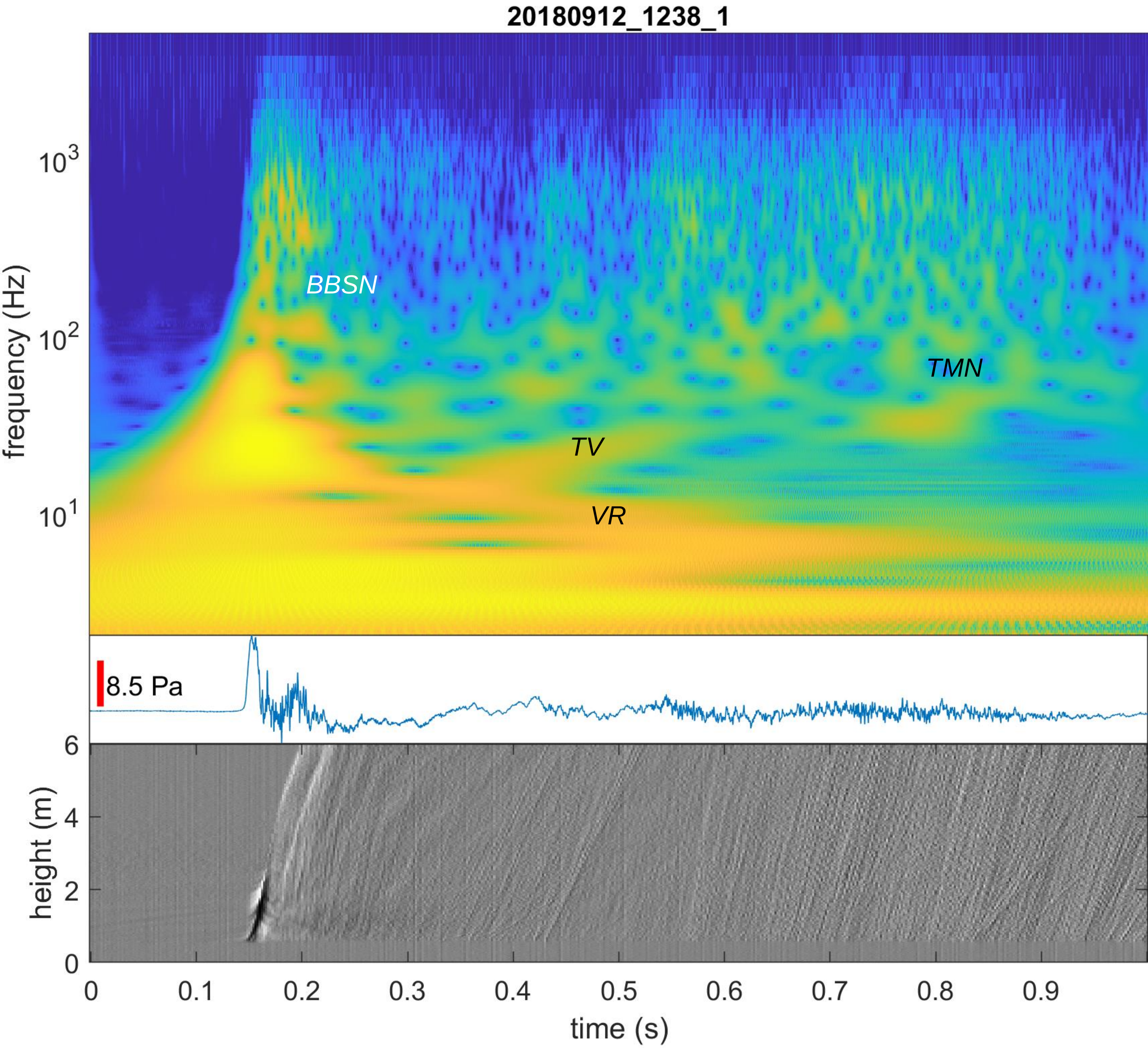
20180912_1238_1
frequency (Hz)
10^3
10^2
10^1
BBSN
TMN
TV
VR
8.5 Pa
height (m)
6
4
2
0
0
0.1
0.2
0.3
0.4
0.5
0.6
0.7
0.8
0.9
time (s)

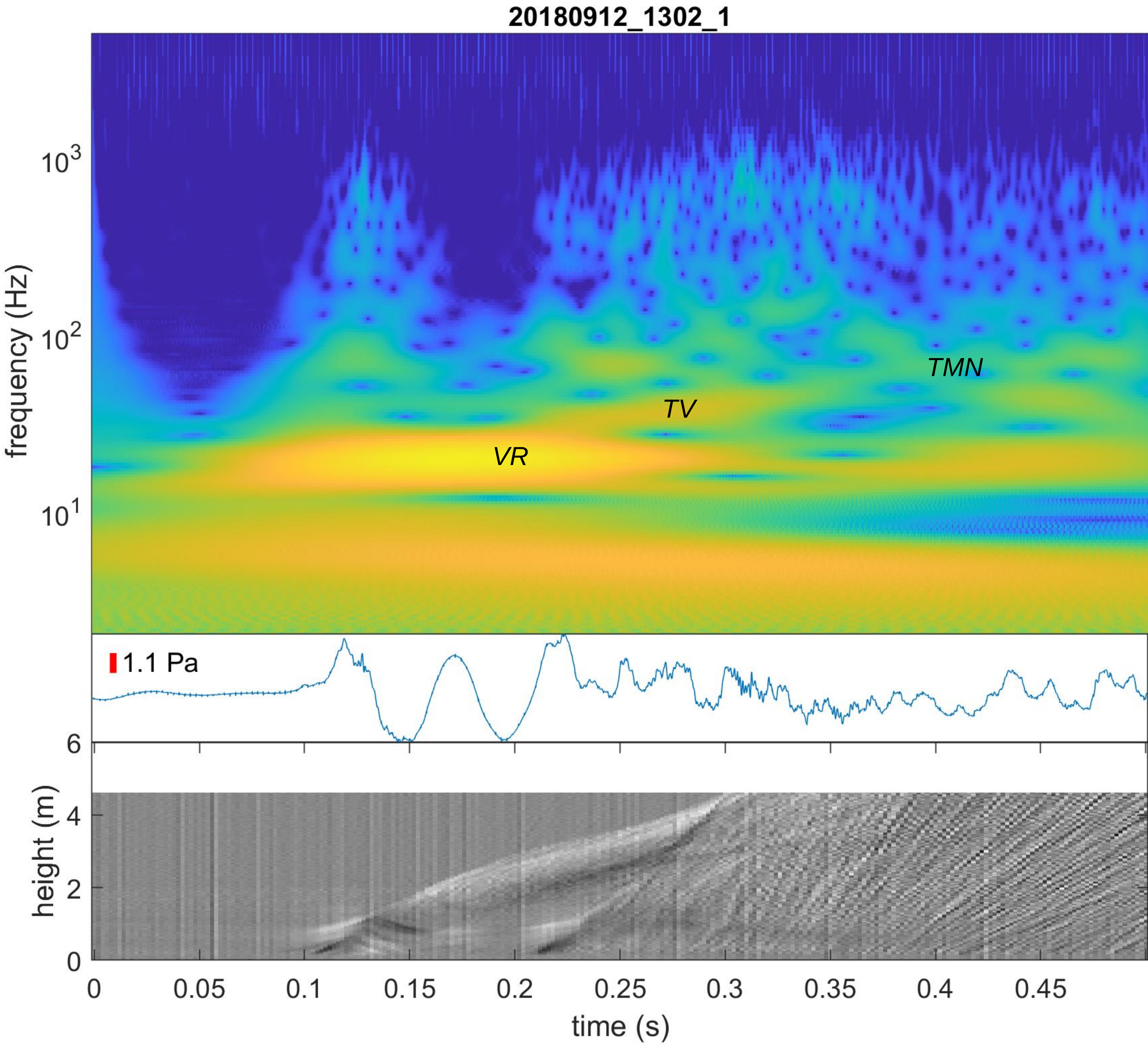
20180912_1302_1
frequency (Hz)
10^3
10^2
10^1
VR
TV
TMN
1.1 Pa
height (m)
6
4
2
0
0
0.05
0.1
0.15
0.2
0.25
0.3
0.35
0.4
0.45
time (s)

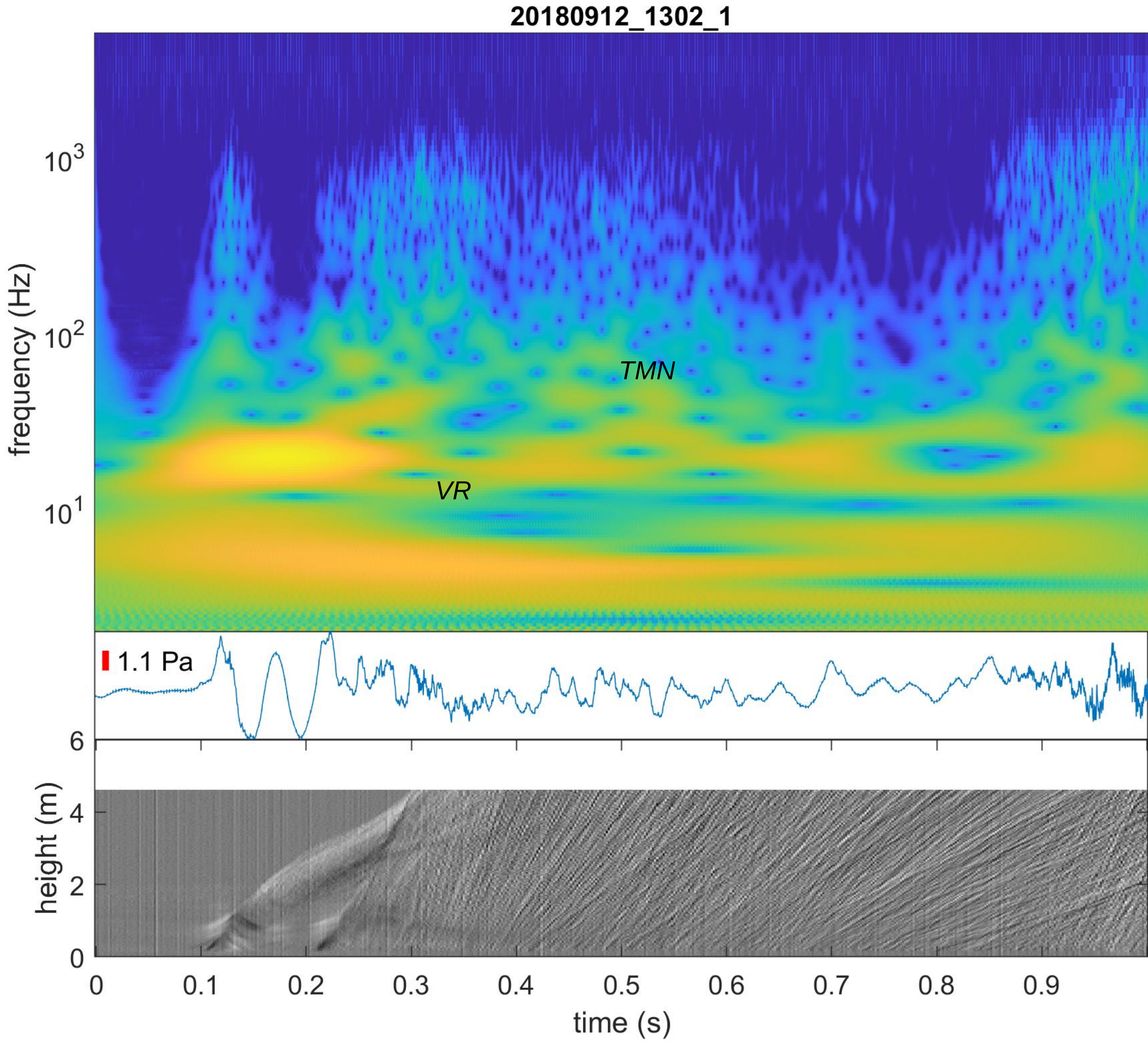

20180912_1302_1
frequency (Hz)
10^3
10^2
10^1
TMN
VR
1.1 Pa
height (m)
6
4
2
0
0
0.1
0.2
0.3
0.4
0.5
0.6
0.7
0.8
0.9
time (s)

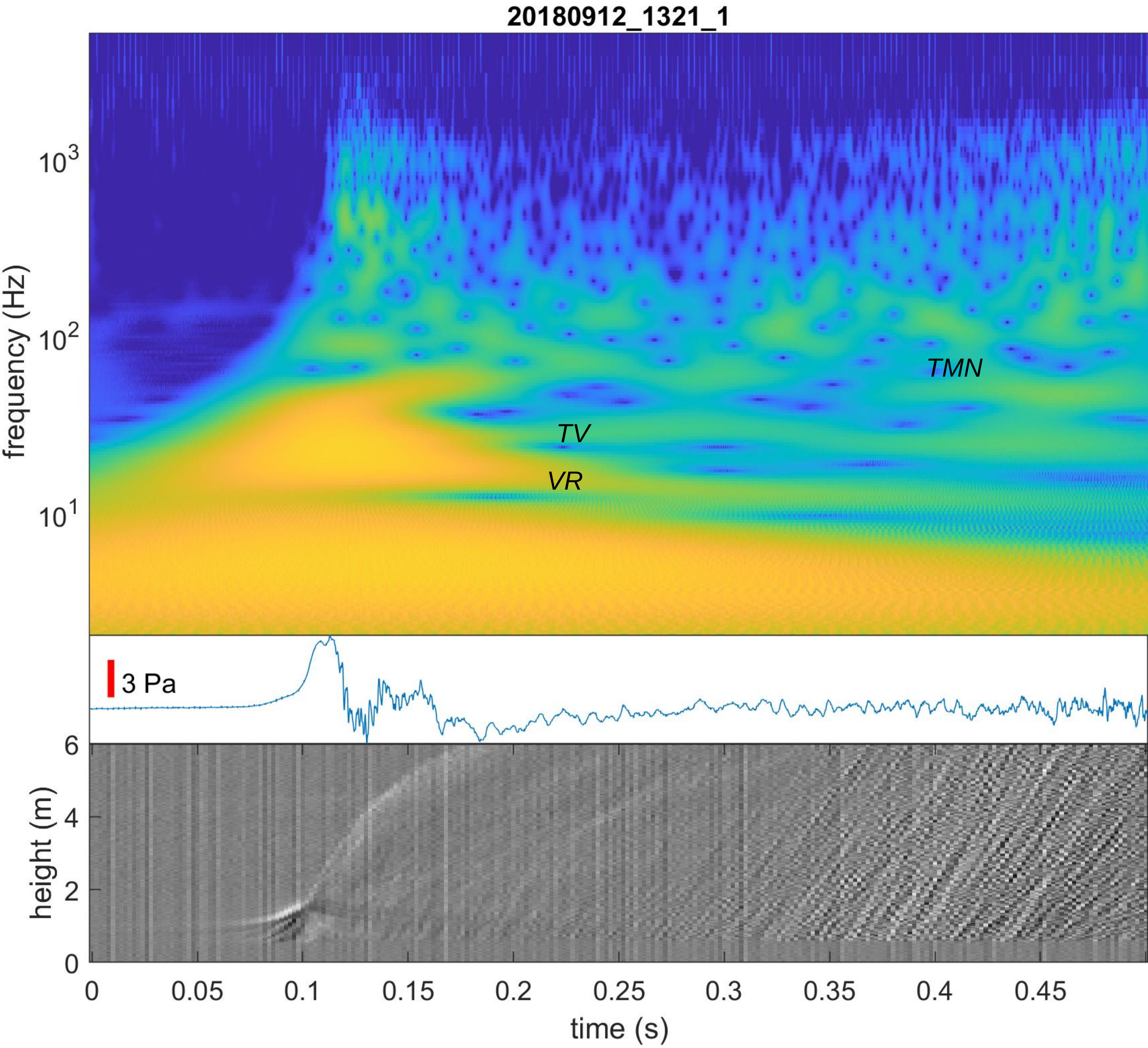
20180912_1321_1
frequency (Hz)
10^3
10^2
10^1
TMN
TV
VR
3 Pa
height (m)
6
4
2
0
0.05
0.1
0.15
0.2
0.25
0.3
0.35
0.4
0.45
time (s)

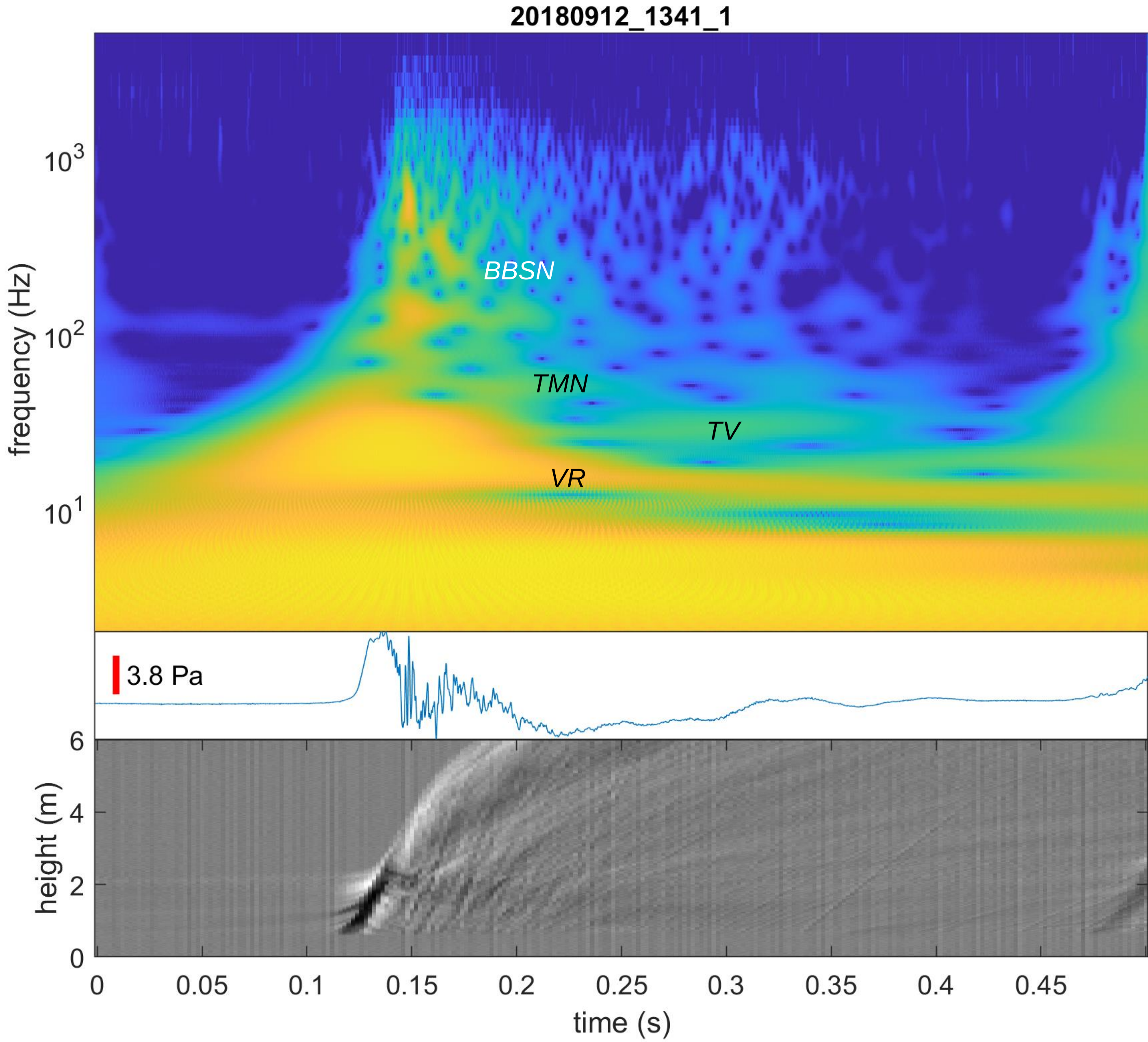

20180912_1341_1
BBSN
TMN
TV
VR
10^3
10^2
10^1
frequency (Hz)
3.8 Pa
6
4
2
0
height (m)
0
0.05
0.1
0.15
0.2
0.25
0.3
0.35
0.4
0.45
time (s)

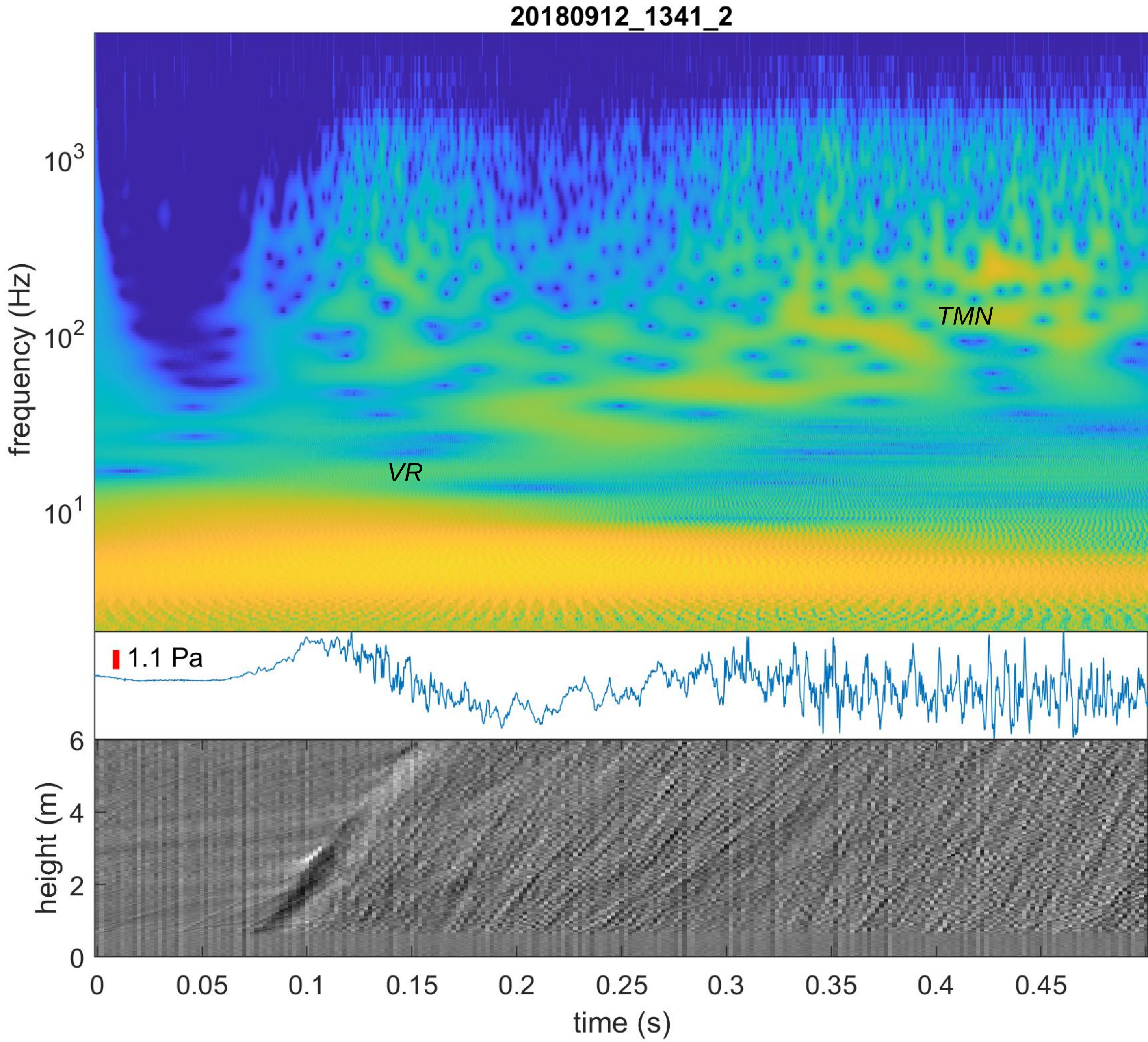
20180912_1341_2
frequency (Hz)
10^3
10^2
10^1
TMN
VR
1.1 Pa
height (m)
6
4
2
0
0
0.05
0.1
0.15
0.2
0.25
0.3
0.35
0.4
0.45
time (s)

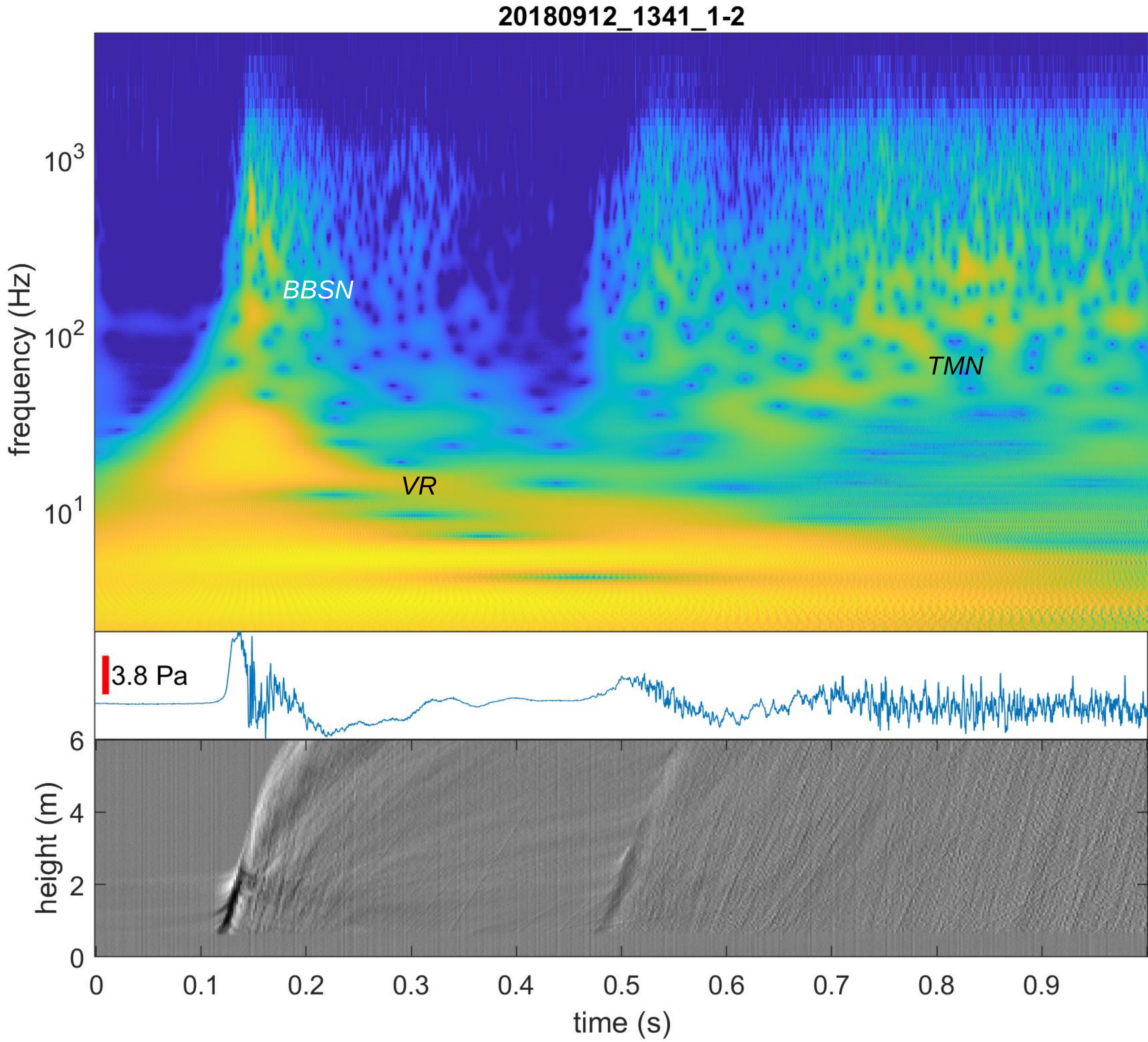

20180912_1341_1-2
frequency (Hz)
10^3
10^2
10^1
BBSN
TMN
VR
3.8 Pa
height (m)
6
4
2
0
0.1
0.2
0.3
0.4
0.5
0.6
0.7
0.8
0.9
time (s)

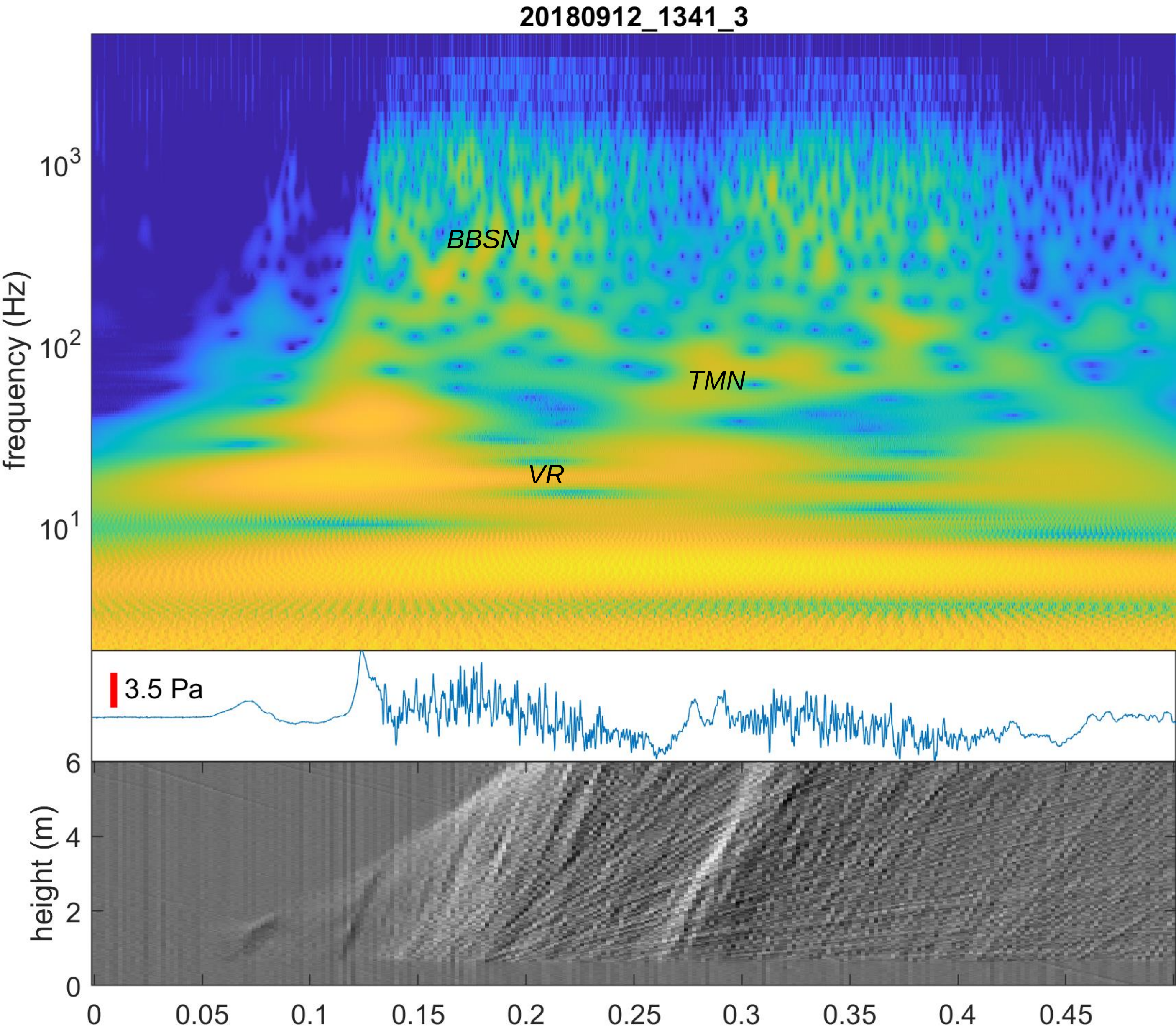
20180912_1341_3
frequency (Hz)
10^3
10^2
10^1
BBSN
TMN
VR
3.5 Pa
height (m)
6
4
2
0
0.05
0.1
0.15
0.2
0.25
0.3
0.35
0.4
0.45
time (s)

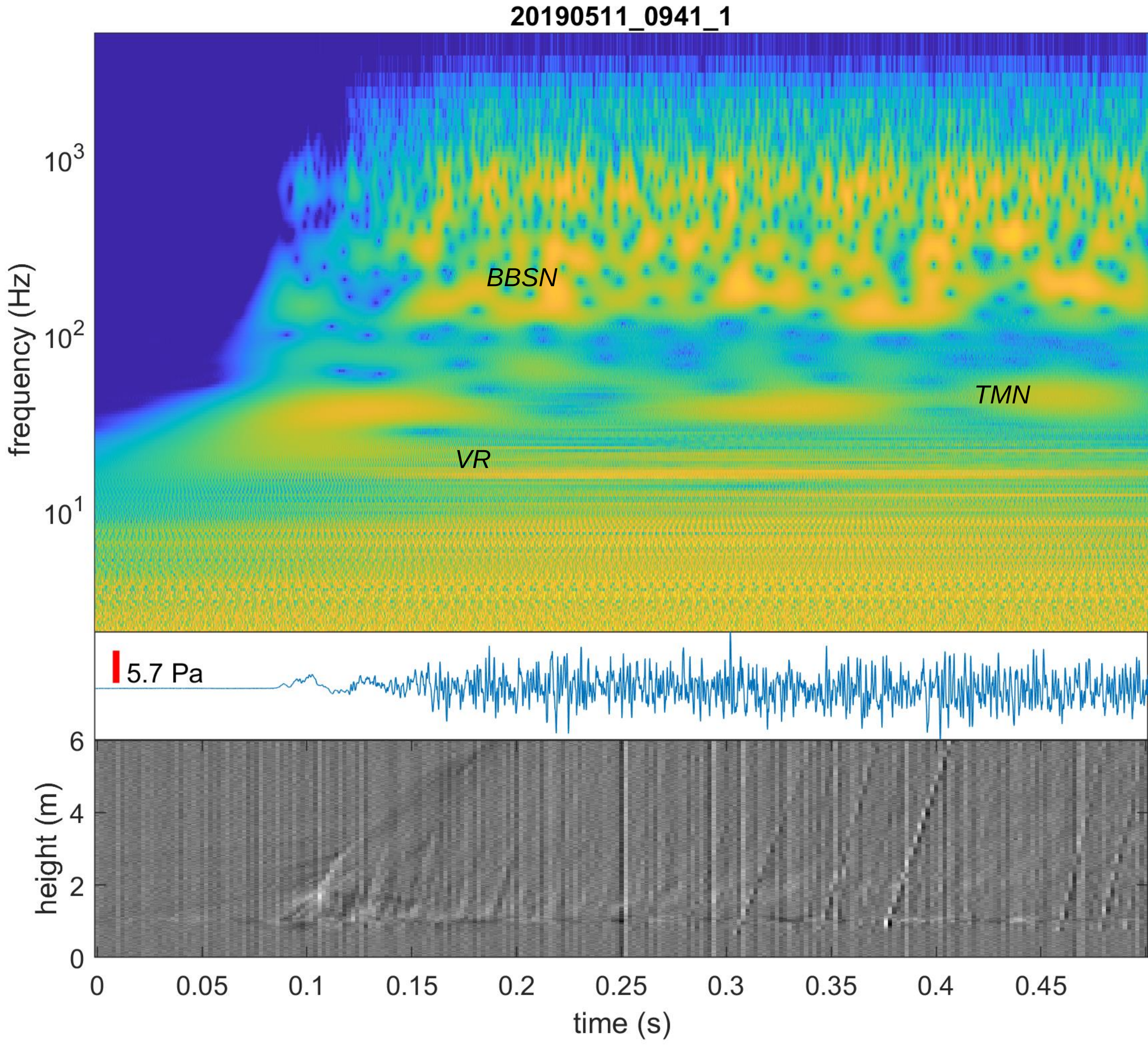

20190511_0941_1
frequency (Hz)
BBSN
TMN
VR
5.7 Pa
height (m)
time (s)

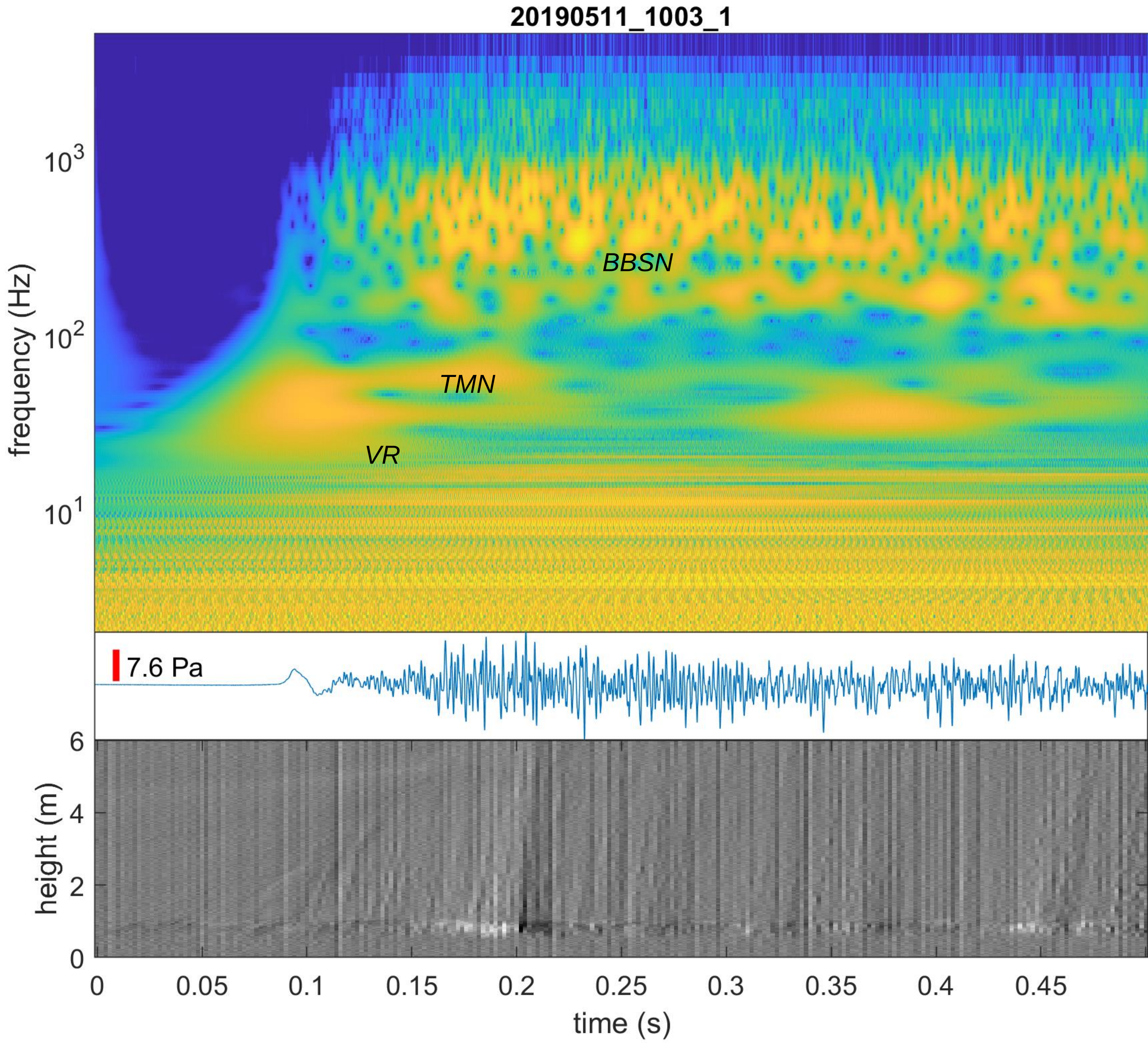
20190511_1003_1
frequency (Hz)
10^3
10^2
10^1
BBSN
TMN
VR
7.6 Pa
height (m)
6
4
2
0
0
0.05
0.1
0.15
0.2
0.25
0.3
0.35
0.4
0.45
time (s)

20190511_1048_1

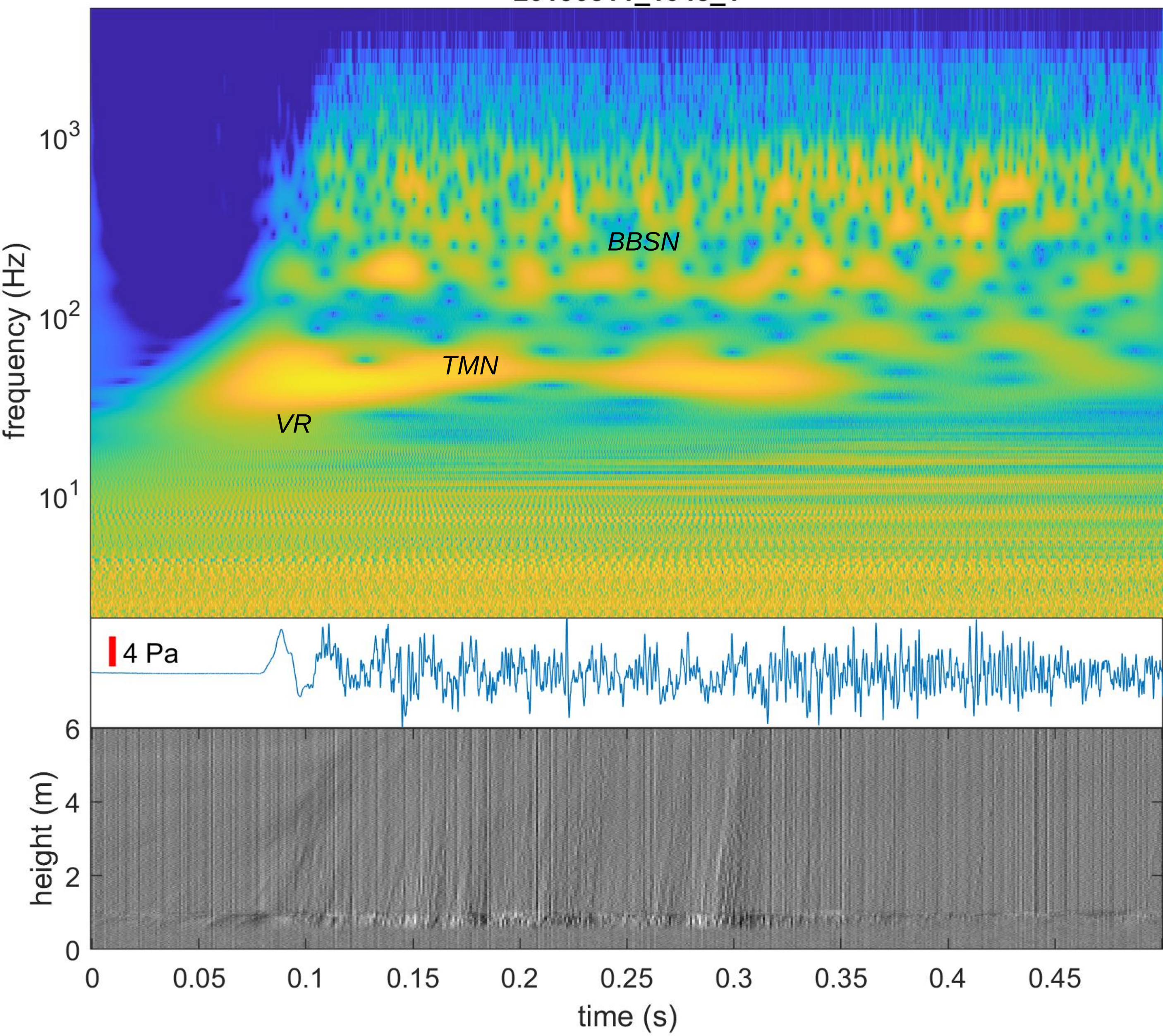

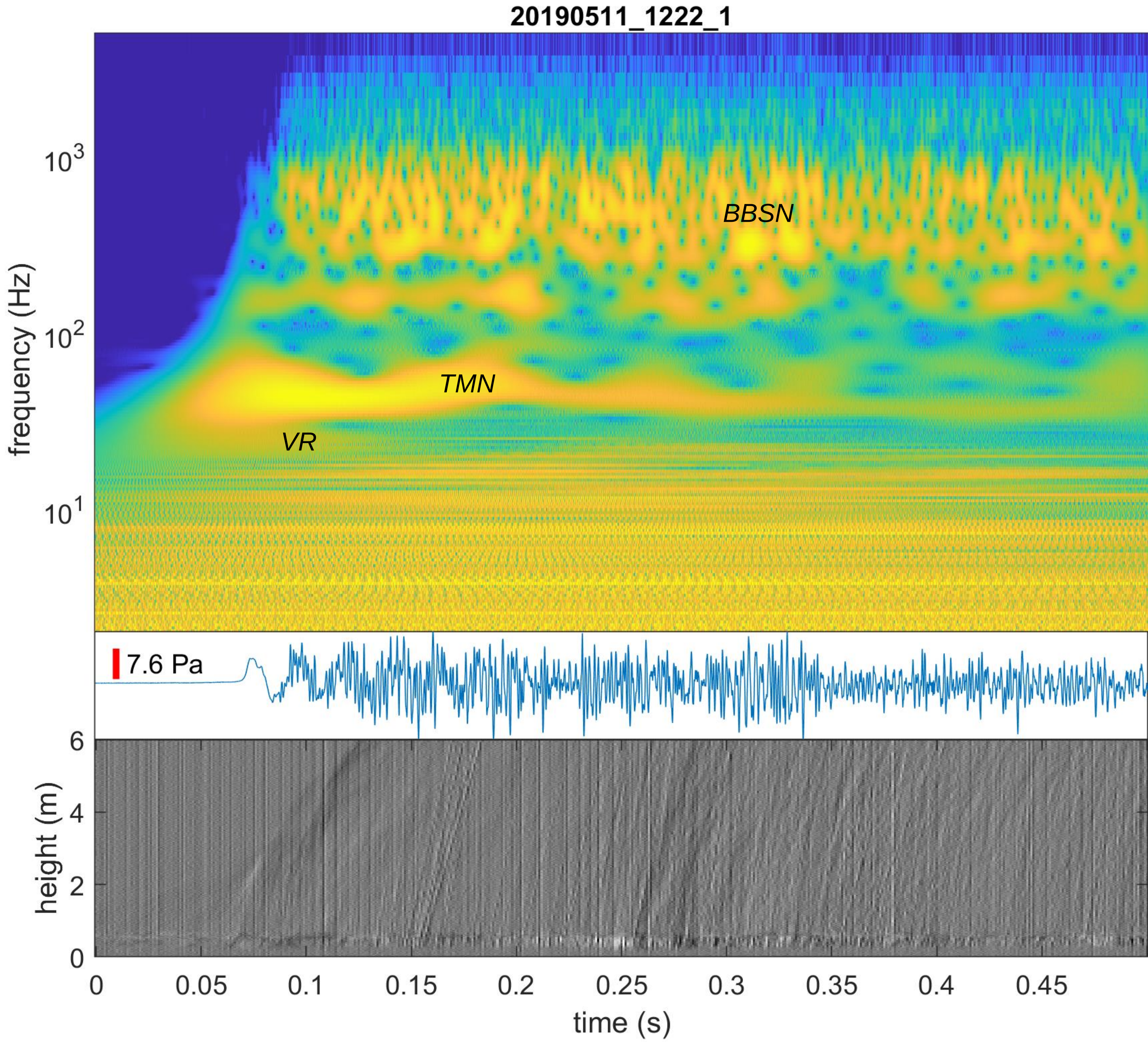

20190511_1222_1
frequency (Hz)
10^3
10^2
10^1
BBSN
TMN
VR
7.6 Pa
height (m)
6
4
2
0
0
0.05
0.1
0.15
0.2
0.25
0.3
0.35
0.4
0.45
time (s)

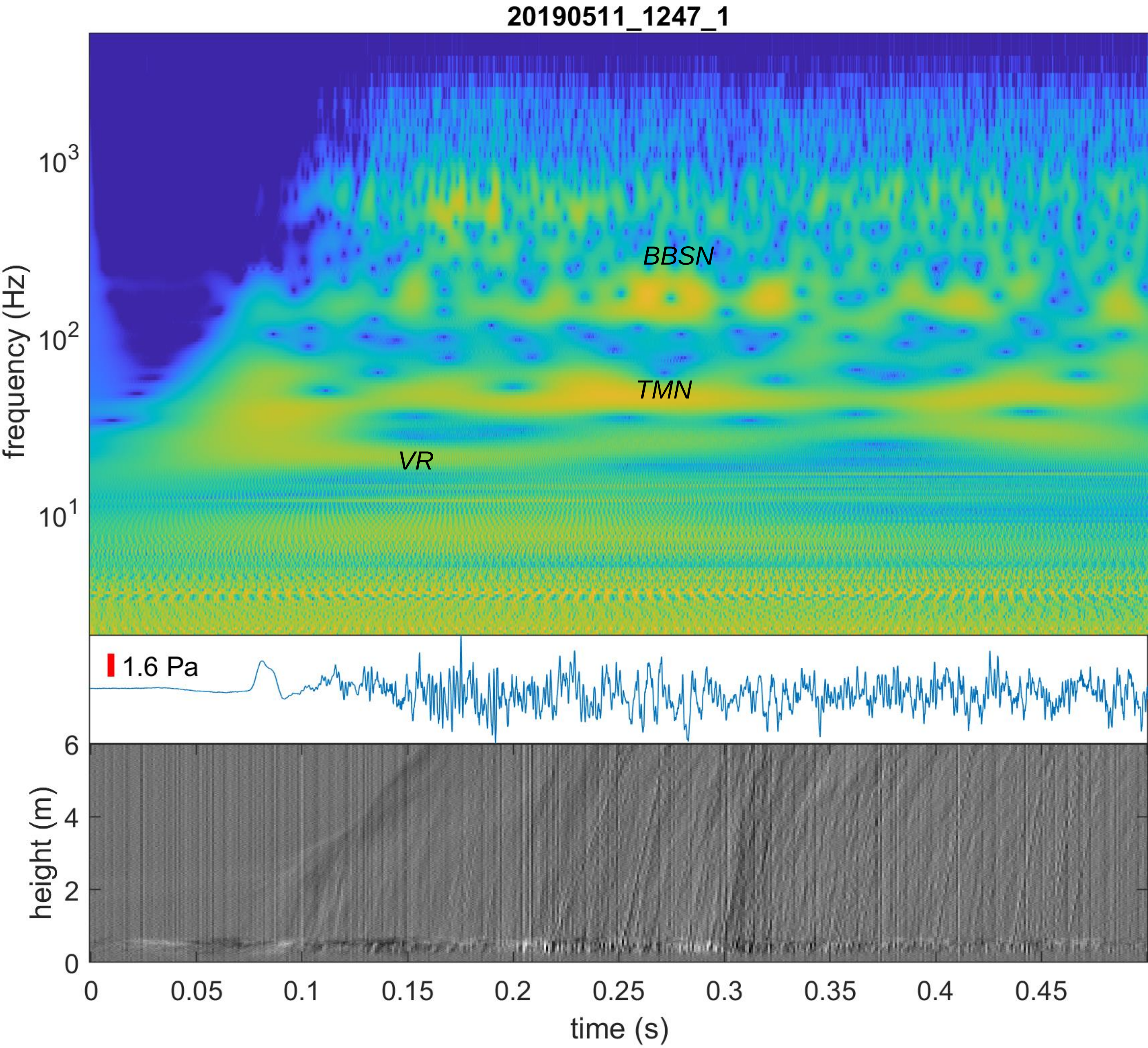
20190511_1247_1
frequency (Hz)
10^3
10^2
10^1
BBSN
TMN
VR
1.6 Pa
height (m)
6
4
2
0
time (s)
0
0.05
0.1
0.15
0.2
0.25
0.3
0.35
0.4
0.45

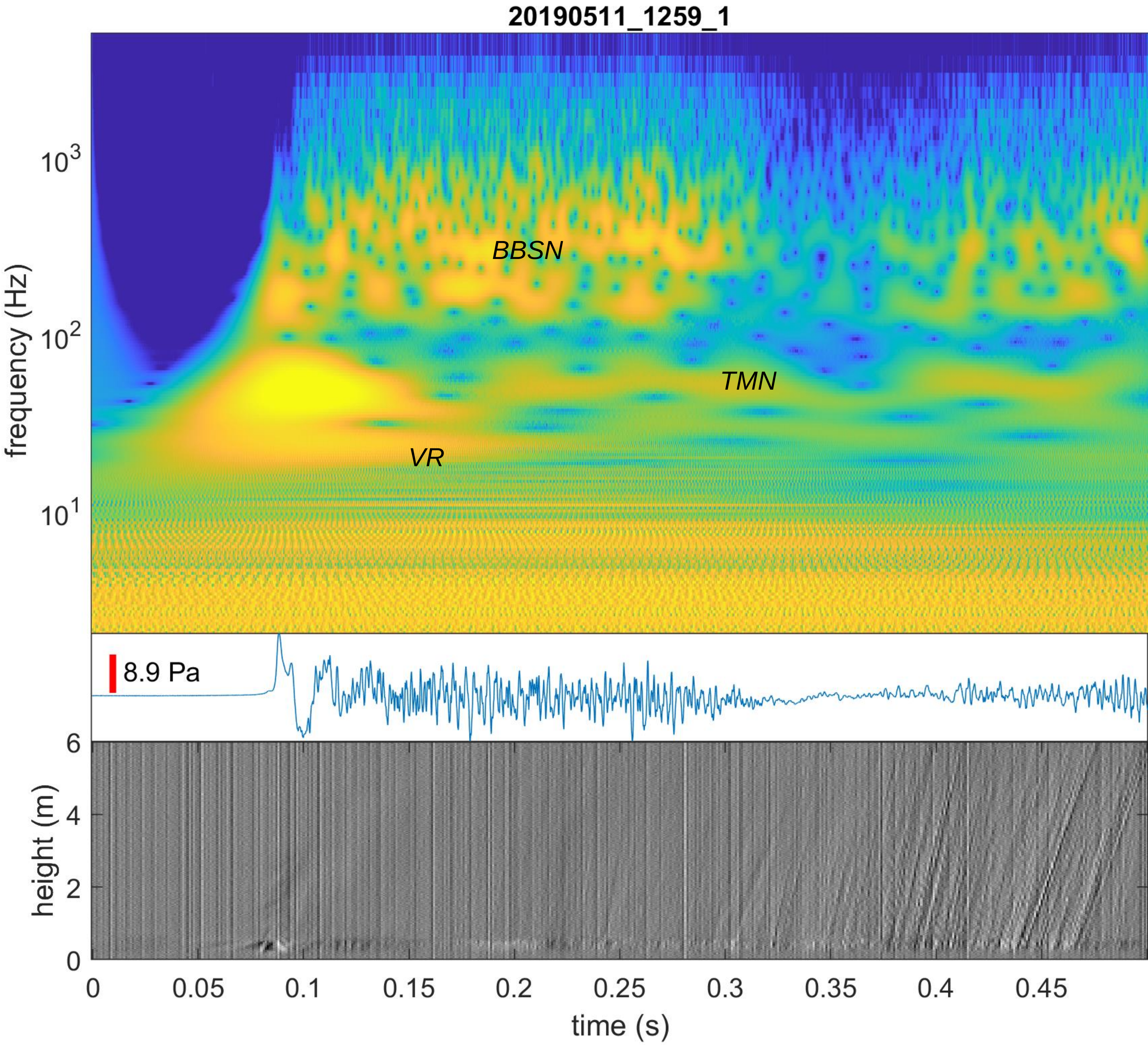

20190511_1259_1
frequency (Hz)
10^3
10^2
10^1
BBSN
TMN
VR
8.9 Pa
height (m)
6
4
2
0
0
0.05
0.1
0.15
0.2
0.25
0.3
0.35
0.4
0.45
time (s)

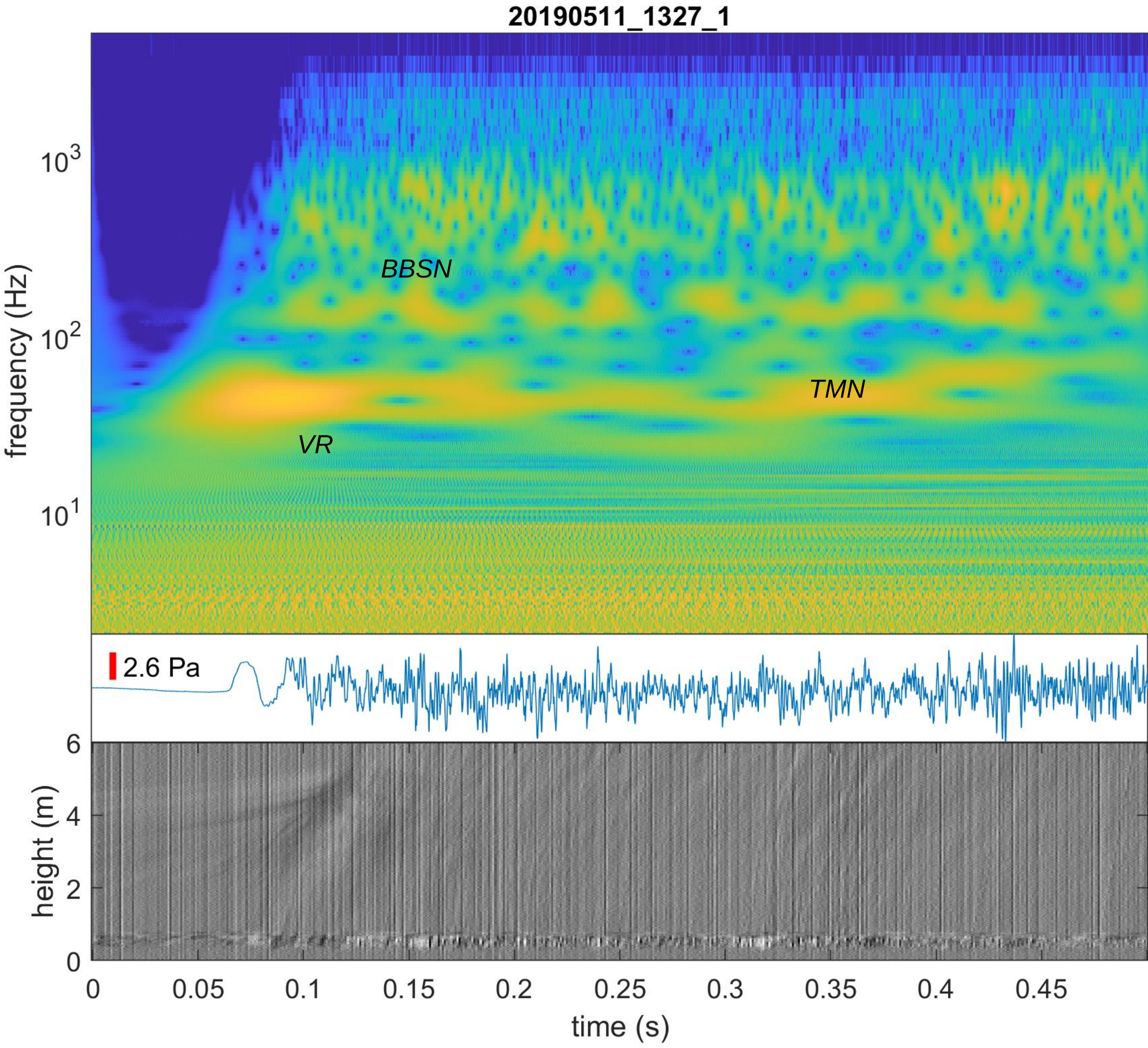
20190511_1327_1
frequency (Hz)
10^3
10^2
10^1
BBSN
TMN
VR
2.6 Pa
height (m)
6
4
2
0
0
0.05
0.1
0.15
0.2
0.25
0.3
0.35
0.4
0.45
time (s)
